# Dynamin‐Related Protein 1‐Dependent Disruption of Mitochondrial Homeostasis Drives Blue Light‐Induced Epithelial‐Mesenchymal Transition in Retinal Aging

**DOI:** 10.1111/acel.70416

**Published:** 2026-02-16

**Authors:** Zhi‐Yuan Li, Dashuang Yang, Yongxia Huang, Yintian Li, Tianyun Zhao, Ying Xu

**Affiliations:** ^1^ Guangdong‐Hongkong‐Macau Institute of CNS Regeneration, Key Laboratory of CNS Regeneration (Jinan University)‐Ministry of Education, Guangdong Key Laboratory of Non‐Human Primate Research Jinan University Guangzhou P. R. China; ^2^ Department of Anesthesiology, Guangzhou Women and Children's Medical Center Guangzhou Medical University Guangzhou China

**Keywords:** age‐related macular degeneration, Drp1, epithelial‐mesenchymal transition, mitochondrial dynamics, oxidative stress, retinal pigment epithelial

## Abstract

Age‐related macular degeneration (AMD) stands as a leading cause of blindness in the elderly, yet the fundamental aging processes that underpin its pathogenesis remain incompletely defined. The dysfunction of retinal pigment epithelial (RPE) cells is a central event in AMD, a process that shares key hallmarks with broader cellular aging, particularly the progressive decline in mitochondrial function. In this study, we investigated how a common environmental stressor—blue light—triggers a key pathological transformation, epithelial‐mesenchymal transition (EMT), in RPE cells by specifically disrupting mitochondrial dynamics, a core pillar of cellular aging. Using an in vitro model of human RPE cells, we demonstrated that blue light exposure induces a marked shift in mitochondrial dynamics towards excessive fission. This imbalance directly resulted in mitochondrial dysfunction, elevated oxidative stress, and served as the critical driver for the initiation of EMT. Importantly, pharmacological inhibition of the mitochondrial fission GTPase Dynamin‐related protein 1 (Drp1) with Mdivi‐1 effectively restored mitochondrial network homeostasis, rescued mitochondrial function, and fully reversed the EMT phenotype. These findings were corroborated in a mouse model of blue light‐induced retinal damage, where Drp1 inhibition successfully preserved retinal light responses, mitigated structural degeneration, and slowed disease progression. Our study demonstrates that Drp1‐mediated excessive mitochondrial fission drives EMT in RPE cells under blue light, linking this mechanism to AMD progression. Consequently, targeting mitochondrial dynamics to maintain cellular homeostasis emerges as a promising and broadly applicable geroscience‐based strategy for mitigating age‐related tissue dysfunction.

## Graphical abstract

Blue light causes retinal injury by inducing excessive mitochondrial fission and epithelial‐mesenchymal transition in retinal pigment epithelial cells. Therapeutic targeting of Drp1 rescues this damage, restoring mitochondrial homeostasis and retinal integrity.

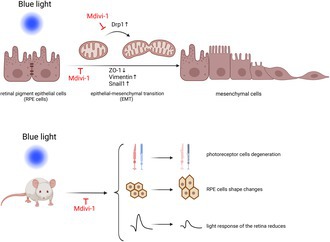

**Abbreviations**
AD: Alzheimer's disease
AMD: age‐related macular degeneration
BSA: bovine serum albumin
DHE: dihydroethidium
DMEM/F12: Dulbecco's Modified Eagle Medium/Nutrient Mixture F12 Ham
Drp1: dynamin‐related protein 1
EMT: epithelial‐mesenchymal transition
ERG: electroretinography
GAPDH: glyceraldehyde 3‐phosphate dehydrogenase
MFN2: Mitofusin‐2
mtDNA: mitochondrial DNA
nAMD: neovascualr AMD
OMA1: overlapping with the m‐AAA protease 1 homolog
ONL: outer nuclei layer
OPA1: optic atrophy 1
PBS: phosphate buffered solution
PD: parkinson's disease
PFA: paraformaldehyde
RIPA: radioimmunoprecipitation assay
ROS: reactive oxygen species
RPE: retinal pigment epithelium
RT‐qPCR: quantitative reverse transcription polymerase chain reaction
SDS‐PAGE: Sodium Dodecyl Sulfate Polyacrylamide Gel Electrophoresis
TBS: Tris buffered saline
TBST: Tris‐buffered saline with 0.1%Tween
TOMM20: translocase of outer mitochondrial membrane 20 homolog
ZO‐1: zonula occludens‐1

## 1 Background

Age‐related macular degeneration (AMD) is a leading cause of irreversible blindness in people over 50, affecting nearly 200 million individuals worldwide and projected to reach 288 million by 2040 (Hanus et al. 2015; Fleckenstein et al. 2021). Notably, over 90% of AMD cases present as dry AMD, characterized by progressive dysfunction and loss of the retinal pigment epithelium (RPE) (Blasiak et al. 2022). Until recently, there was no effective therapy for this condition. This changed with the approval of the C3 inhibitor pegcetacoplan (Trivizki et al. 2025) and the C5 inhibitor avacincaptad pegol (Kang 2023) for the treatment of geographic atrophy, the advanced form of dry AMD. Although these drugs can slow the progression of atrophy, they require frequent intravitreal injections, offer limited visual improvement, and are associated with a high incidence of side effects (Shen et al. 2026). Therefore, elucidating the underlying mechanisms of RPE degeneration and exploring new therapeutic strategies for AMD remain critically important (Bresciani et al. 2024).

Among the proposed mechanisms, the epithelial–mesenchymal transition (EMT) in RPE cells has emerged as a key contributor to dry AMD pathology (Shu et al. 2020; Wang et al. 2025). During EMT, epithelial cells lose polarity and tight junctions and acquire mesenchymal features (Chen et al. 2025). Aging (Gao et al. 2022), oxidative stress (Yang et al. 2024), and exposure to bright light (Iriyama et al. 2008) can all trigger EMT‐like changes in RPE cells, and both experimental and clinical studies have linked RPE EMT to AMD progression (Shu et al. 2020; Chowdhury et al. 2025). Inhibiting EMT alleviates RPE dysfunction and retinal pathology in several dry AMD models, suggesting its potential as a therapeutic target (Yang et al. 2024; Hui et al. 2024; Ghosh et al. 2018).

However, the upstream drivers of EMT in RPE degeneration remain unclear. Recent evidence highlights the role of mitochondrial dynamics—particularly the balance between fission and fusion—in regulating cellular differentiation, metabolic homeostasis, and stress responses (Tan et al. 2020; Buonfiglio et al. 2024; Lugassy et al. 2025). Disruption of this balance impairs mitochondrial function and increases ROS (Shen et al. 2023; Han et al. 2023), features also seen in AMD RPE tissues (Yu et al. 2020; Chen et al. 2014) and in blue‐light–injured RPE cells (Han et al. 2023; Alaimo et al. 2019). Excessive mitochondrial fission has been shown to promote EMT in multiple cell types (Feng et al. 2025; Ghosh et al. 2023; Zhao et al. 2020), and blocking fission suppresses EMT‐related phenotypes (Chen et al. 2025; Han et al. 2023; Ashraf and Kumar 2022). These observations raise the possibility that deregulated mitochondrial dynamics may contribute to RPE EMT during AMD development, yet this connection remains insufficiently explored.

In this study, we investigated the role of mitochondrial dynamics in RPE EMT using a blue‐light–induced dry AMD model. We examined whether inhibiting mitochondrial fission could preserve RPE structure and retinal function, and we evaluated the downstream impact on EMT markers, mitochondrial integrity, and oxidative stress. Our findings provide mechanistic insights into how mitochondrial dysfunction may drive EMT in dry AMD and support mitochondrial dynamics as a potential therapeutic target.

## 2 Materials and Methods

### 2.1 Cell Culture and Light Injury

The human RPE cell line ARPE‐19, obtained from Aier Eye Hospital, was cultured at 37°C in a 5% CO2 and humidified environment. The cells were maintained in Dulbecco's Modified Eagle Medium/Nutrient Mixture F12 Ham (DMEM/F12, Shanghai XP Biomed Ltd., China), supplemented with 10% heat‐inactivated fetal bovine serum (Tiktaalik, Guangzhou, China) and 1% penicillin–streptomycin (Tiktaalik, Guangzhou, China). Once the cells reached 80%–90% confluence, they were trypsinized with 0.25% Trypsin–EDTA (Gibco, Canada) and plated for subsequent experiments.

To establish a blue light injury model, an LED array (450–480 nm) was placed in the incubator, with a luminance of 5000 ± 200 lx. Cells at a density of 1 × 10^5^ cells/mL were cultured for 24 h before being exposed to blue light for an additional 48 h. Control cells were shielded from blue light using tin foil. During the 48‐h exposure, the incubator temperature was maintained at 37°C using a thermometer.

To investigate whether Drp1‐mediated mitochondrial fission is involved in light‐induced damage, ARPE‐19 cells were pretreated with 10 μM or 20 μM Mdivi‐1 (a specific Drp1 inhibitor) for 1 h before light exposure. Following pretreatment, the culture medium was replaced with DMEM/F12 for the remainder of the experiment.

### 2.2 Cell Viability Assay

ARPE‐19 cells were seeded at a density of 1 × 10^5^/mL in a 48‐well cell culture plate and cultured at 37°C in a 5% CO_2_ incubator for 24 h to allow for cell adhesion. The culture medium was then replaced with fresh DMEM/F12 medium containing different concentrations of Mdivi‐1 (ranging from 5 to 40 μM). After 48 h of incubation, cell viability was assessed using the CCK‐8 assay (Beyotime Biotechnology, China). Following the operating procedures of the CCK8 assay kit, the treated cells were co‐incubated with culture medium containing 10% CCK8 reagent for 1 h, and then the absorbance was measured at 450 nm using a microplate reader. Cell viability is calculated by comparing the absorbance values of each group.

### 2.3 Reactive Oxygen Species (ROS) Detection

To quantify ROS levels in ARPE‐19 cells, we used the superoxide anion fluorescent probe Dihydroethidium (DHE, Beyotime Biotechnology, China). Following 48 h of blue light exposure, cells were incubated with 10 μM DHE for 30 min at 37°C, as per the manufacturer's instructions. After two washes with Hank's Balanced Salt Solution buffer (Gibco, Canada), the fluorescence signal of DHE was observed and photographed using a live cell workstation (Zeiss, Germany). ImageJ software was then used to quantify the fluorescence intensity, reflecting the ROS levels.

### 2.4 Animals and Blue Light Damage Model

Ten‐week‐old male Balb/c mice, sourced from Liaoning Changsheng Biotechnology Co. Ltd. (China), were housed in a controlled environment with a 12‐h light/dark cycle (50 lx) and ad libitum access to food and water. Animal handling adhered to NIH guidelines and those of the Institutional Animal Care and Use Committee of Jinan University (Guangzhou, China), under license number: SYXK (Guangdong) 2022‐0174. Use and care of mice followed the guidelines of the Institutional Animal Care and Use Committee of Jinan University (IACUC‐20241018‐06, Guangzhou, China). All operations in the experiment followed the regulations of the NIH and the Experimental Animal Ethics Committee of Jinan University.

The mice were randomly assigned to two groups: a control group and a blue light exposure group, with at least three animals per group. The light exposure box featured a blue LED array (10,000 lx when on) and foil‐covered walls and bottom to ensure uniform lighting. The blue light injury group underwent anesthesia with tribromoethanol, pupil dilation with cotrimoxazole eye drops, and direct exposure to intense blue light (20,000 lx) for 1 h. Subsequently, they were placed in the box with LEDs on for 11 h and off for 12 h daily for a week. The control group was housed in the same box but exposed to standard room light during the LED‐on period.

Following the week of blue light exposure, visual behavioral tests were conducted the next day, followed by overnight dark adaptation for electroretinogram recordings the subsequent day. All animals were euthanized by cervical dislocation, and tissues were collected for further analysis.

For the treatment of animals with the Drp1 inhibitor Mdivi‐1, mice received intraperitoneal injections of Mdivi‐1 solution 1 h before each blue light exposure session throughout the week. Doses including 10, 20, and 50 mg/kg body weight were first tested for safety on normal mice; then the 20 mg/kg dose was chosen for the treatment of blue light injured mice. Note that the drug concentrations are reported in μM for in vitro experiments and as administered dose (mg/kg) for in vivo experiments, consistent with field standards.

### 2.5 Visual Behavior Test

We employed an optokinetic system to assess mouse visual acuity by monitoring head rotations in response to moving gratings, as previously described (Liu et al. 2021). Mice were positioned on a centrally elevated platform surrounded by a computer screen displaying vertically rotating sinusoidal gratings. These gratings were programmed using MATLAB (version 8.0, MathWorks, Natick, MA, USA) with 100% contrast, moving at 12 cycles per second, and gradually increasing in spatial frequency from 0.1 to 0.6 cycles per degree. The animals instinctively tracked the gratings with their heads (optokinetic reflex) when visible. Head movements were recorded via video, and the maximum spatial frequency eliciting an optokinetic response was manually noted to indicate the mice's visual acuity.

### 2.6 Electroretinography (ERG)

To evaluate the light response of the retina, Balb/C mice were dark‐adapted for 12 h and subsequently underwent ERG experiments using a RETI scanning system (Roland Consult, Brandenburg, Germany) as we previously described (Liu et al. 2021). Under anesthesia with tribromoethanol (0.2 mL/10 g of a 1.25% solution) and dim red light conditions, their pupils were dilated with 0.5% tropicamide. A gold‐plated wire loop electrode in contact with the corneal surface served as the active electrode, while stainless steel needle electrodes inserted through the caudal and retroauricular skin acted as ground and reference leads, respectively. Dark‐adapted mice were stimulated with green light flashes of varying intensities (0.01, 0.1, and 3.0 cd.s/m^2^). The recorded signals, filtered at 50 Hz, were analyzed using RETIport software. The a‐wave amplitude was measured from baseline to the first negative peak, and the b‐wave amplitude from the a‐wave trough to the subsequent positive peak. Responses from both eyes of each mouse were averaged to obtain a single data point.

### 2.7 Immunofluorescence Staining

For immunofluorescent staining of ARPE‐19, after blue light exposure, the cells were fixed in 4% paraformaldehyde for 30 min, then incubated in a blocking solution containing 10% donkey serum protein and 0.2% Triton‐X reagent in phosphate buffered solution (PBS, Beyotime Biotechnology, China). Cells were then incubated with the primary antibody in the blocking buffer overnight at 4°C and washed three times with PBS the next day. Cells were then incubated with secondary antibody and DAPI (17984‐24, Electron Microscopy Sciences, 1:1000) in PBS for 2 h at room temperature protected from light, followed by washing in PBS solution three times. Cells were then sealed on a slide with an anti‐fluorescence quenching sealing solution.

For immunofluorescence staining of retinal sections, eyes were removed immediately after sacrificing the mice, then fixed in 4% paraformaldehyde (PFA, Beyotime Biotechnology, China) for 1 h, and immersed in 30% sucrose at 4°C for 24 h. The eyes were subsequently embedded in OCT embedding agent (BL557A, Biosharp, China) and stored at −80°C till sectioning. Using a frozen sectioning machine, sections were cryo‐sectioned longitudinally along the optic nerve disc at a thickness of 16 μm per section, and stored at −40°C before staining. For immunofluorescence staining, the tissue sections were washed three times with PBS and incubated for 2 h in a blocking solution containing 10% donkey serum protein and 0.3% Triton‐X reagent in PBS. This was followed by overnight incubation at 4°C with the primary antibody, and the next day, the sections were washed three times with PBS, followed by the addition of secondary antibody and DAPI for 2 h of incubation at room temperature protected from light.

For immunofluorescence staining of the whole mount retina, the retina was extracted from the eyeball after fixation by PFA, then incisions were made to flatten the whole‐mount retina before incubating with primary and secondary antibody as described above. For immunostaining of RPE tissue, lens and retina were removed after fixing the eyeball with PFA for 1 h, and then the RPE‐choroid‐sclera complex was flattened by four radial incisions and incubated with primary and secondary antibody as described above.

Primary antibodies used in this study included anti‐rabbit‐Drp1 (ab184247, abcam, 1:500), anti‐rabbit‐Vimentin (R1380‐6, Huabio, 1:1000), anti‐rabbit‐ZO‐1 (Zonula occludens‐121773‐1‐AP, Proteintech, 1:1000), anti‐rabbit‐Snail1 (ER1706‐22, Huabio, 1:100), anti‐TOMM20 (Translocase of outer mitochondrial membrane 20 homolog, sc‐17764, Santa Cruz Biotechnology, 1:1000), anti‐rabbit‐Opsin (ABN1660, Sigma‐Aldrich, 1:1000), and anti‐mouse‐Rhodopsin (AB_2178961, Millipore,1:1000). Secondary antibodies used included anti‐rabbit‐Alexa 647 (A31573, Invitrogen, 1:1000), anti‐rabbit‐Alexa 488 (A‐21206, Invitrogen, 1:1000), anti‐mouse‐Alexa 647 (A‐31571, Invitrogen, 1:1000). DAPI (17984‐24, Electron Microscopy Sciences, 1:1000) was used to stain the nuclei.

### 2.8 Imaging Collection and Processing

To image the ARPE‐19 cells, 2–3 regions (size of 160 × 160μm^2^) on the slide with 5–10 cells per region were selected randomly and photographed using a confocal microscope (Zeiss). For each region, 5–10 cells with intact cell morphology were randomly selected, and the mean fluorescent intensity of Snail or Vimentin per cell (outlined manually) was measured by Image J. Furthermore, the number of cells exhibiting a continuous ZO‐1 outlining was quantified in each region. The total TOMM20‐positive area was measured and then divided by the total cell area to get the ratio. The data from these regions on one slide were averaged to produce one sample of data.

For immunofluorescence staining of retinal sections, regions (size of 320 × 320 μm^2^) from 500, 1000, and 1500 μm on each side of the optic nerve center were photographed by confocal microscope. The thickness of the outer nuclei layer (ONL, where somas of photoreceptor locate) and rhodopsin+ layer was measured by Image J. The data from the six sites were then averaged to obtain one set of data for this retinal section.

For immunofluorescence staining of retinal whole‐mount, regions (size of 160 × 160 μm^2^) at the center, middle, and peripheral regions from each of the quadrants were collected by a confocal microscopy and number of cone opsin+ outer segment were counted in the full image. Then the data from these regions at various centrifugal distances were averaged to get one data for each mouse. For RPE whole‐mount, regions (size of 160 × 160 μm^2^) were collected randomly by confocal microscopy, and 5 cells in each region were randomly selected to measure the cell area by ImageJ. The data from 6 regions were then averaged as the data for one mouse.

### 2.9 Protein Extraction and Western‐Blotting

To extract and analyze protein from treated ARPE‐19 cells, 100 μL of Radioimmunoprecipitation assay (RIPA, Beyotime Biotechnology, China) buffer containing protease inhibitor was added to 6‐well plates and allowed to stand on ice for 30 min. Cells were then scraped off and transferred to 1.5 mL EP tubes, followed by centrifugation at 14,800 rpm for 30 min at 4°C to collect the supernatant. Protein concentration was determined using a Bicinchoninic Acid assay kit (Beyotime Biotechnology, China), and protein was quantified by mixing with RIPA and 5× SDS‐PAGE loading buffer (Sodium Dodecyl Sulfate Polyacrylamide Gel Electrophoresis, Beyotime Biotechnology, China), heating at 100°C for 10 min, and cooling on ice. The proteins were separated by SDS‐PAGE electrophoresis and transferred to a Polyvinylidene Fluoride Membrane (Millipore, USA), which was blocked with TBS (Tris Buffered Saline, Beyotime Biotechnology, China) containing 5% BSA (Bovine Serum Albumin, Solarbio Science & Technology, China) and 0.1% Tween‐20 (Amresco, USA) for 2 h at room temperature. The membrane was then incubated with the primary antibody overnight at 4°C, washed three times with TBST (Tris‐Buffered Saline with 0.1% Tween), and incubated with HRP‐conjugated secondary antibodies for 90 min at room temperature. After three additional washes with TBST, the reaction product was visualized using an ECL western blot detection kit and a chemical imaging system. Western blot images were exported using Image Lab Software, and band densities were quantitatively assessed using ImageJ.

Primary antibodies were diluted in TBS containing 5% BSA and 0.1% Tween‐20, and secondary antibodies were diluted in 0.1% Tween‐20 TBS. The primary antibodies used were anti‐rabbit‐Drp1 (ab184247, abcam, 1:1000), anti‐rabbit‐Vimentin (R1380‐6, Huabio, 1:5000), anti‐rabbit‐ZO1 (Zonula occludens‐121773‐1‐AP, Proteintech, 1:5000), anti‐rabbit‐Snail1 (ER1706‐22, Huabio, 1:500), anti‐rabbit‐MFN2 (Mitofusin‐2, HA720073, Huabio, 1:1000), anti‐rabbit‐GAPDH (glyceraldehyde‐3‐phosphate dehydrogenase, ET1601‐4, Huabio, 1:3000), anti‐rabbit‐OMA1 (Overlapping with the m‐AAA protease 1 homolog, 17116‐1‐AP, Proteintech, 1:1000), anti‐rabbit‐OPA1 (optic atrophy 1, 27733‐1‐AP, Proteintech, 1:1000), anti‐rabbit‐E‐Cadherin (20874–1‐AP, Proteintech, 1:1000), anti‐rabbit‐Vinculin (13901S, CST, 1:1000), anti‐rabbit‐β‐Tubulin (OB‐RB12M02, OasisBiofarm, 1:2000) and anti‐rabbit‐β‐Actin (20536‐1‐AP, Proteintech, 1:1000). Secondary antibodies used included Rabbit anti‐Goat IgG Secondary Antibody HRP conjugated (L3042, Proteintech, 1:3000).

### 2.10 Quantitative Reverse Transcription Polymerase Chain Reaction (RT‐qPCR)

RT‐qPCR was used to evaluate the expression of genes related to mitochondrial dynamics and epithelial‐mesenchymal transition in the RPE of Balb/c mice after blue light irradiation. The RPE‐choroid complex was first separated from the optic cup using ophthalmic forceps and ophthalmic scissors and immersed in TRIzol reagent (Invitrogen, USA) for RNA extraction according to the manufacturer's instructions. The RNA concentration and purity were determined using a NanoDrop2000c spectrophotometer (Thermo Fisher Scientific). After adjusting the mRNA concentration to a consistent level, cDNA was obtained by reverse transcription using the PrimeScript RT Kit (with gDNA Eraser Perfect Real Time, Takara, Beijing, China). RT‐qPCR primers were designed based on the well‐established primer database, PrimerBank (https://pga.mgh.harvard.edu/primerbank/), and synthesized by Sangon Biotech Co. Ltd. (Shanghai, China). The experiment was performed using TB Green Premix Ex Taq II (Tli RNaseH Plus, Takara) on a LightCycler480 system (Roche, Basel, Switzerland) in strict accordance with the manufacturer's operating procedures. GAPDH was used as the internal reference gene, and the relative quantification of gene expression was determined using the 2^−ΔΔCt^ method (Livak and Schmittgen 2001). The specific oligonucleotide sequences of the primers are provided in Table 1.

**TABLE 1:** The sequences of primers used in this study.

Gene	Species	Sequence (5′−3′)
Drp1	Mouse	Forward: CCTCAGATCGTCGTAGTGGGA Reverse: GTTCCTTGGGAAGAAGGTCC
ZO‐1	Mouse	Forward: GCGCTAAGAGCACAGCAA Reverse: GCCCCTCCTTTTAACACATCAGA
Snail1	Mouse	Forward: CACACGCTGCCTTGTGTCT Reverse: GGTCAGCAAAAGCACGGTT
Vimentin	Mouse	Forward: CGTCCACACGCACCTACAG Reverse: GGGGGATGAGGAATAGAGGCT
GAPDH	Mouse	Forward: CATGGCCTTCCGTGTTCCTA Reverse: CCTGCTTCACCACCTTCTTGAT

### 2.11 Statistical Analysis

All data are expressed as mean ± SD, and all statistical analyses were performed using Prism 7 (GraphPad Software, San Diego, CA, USA). Student *t*‐test, one‐way or two‐way analysis of variance (ANOVA), followed by post hoc multiple comparison, was used for the comparison between two or multiple groups. The scattered dot inside each graph in the figures represents an individual cell well or animal tested in each group.

## 3 Results

### 3.1 Blue Light Induces Mitochondrial Fission and Oxidative Stress in RPE Cells

**FIGURE 1:**
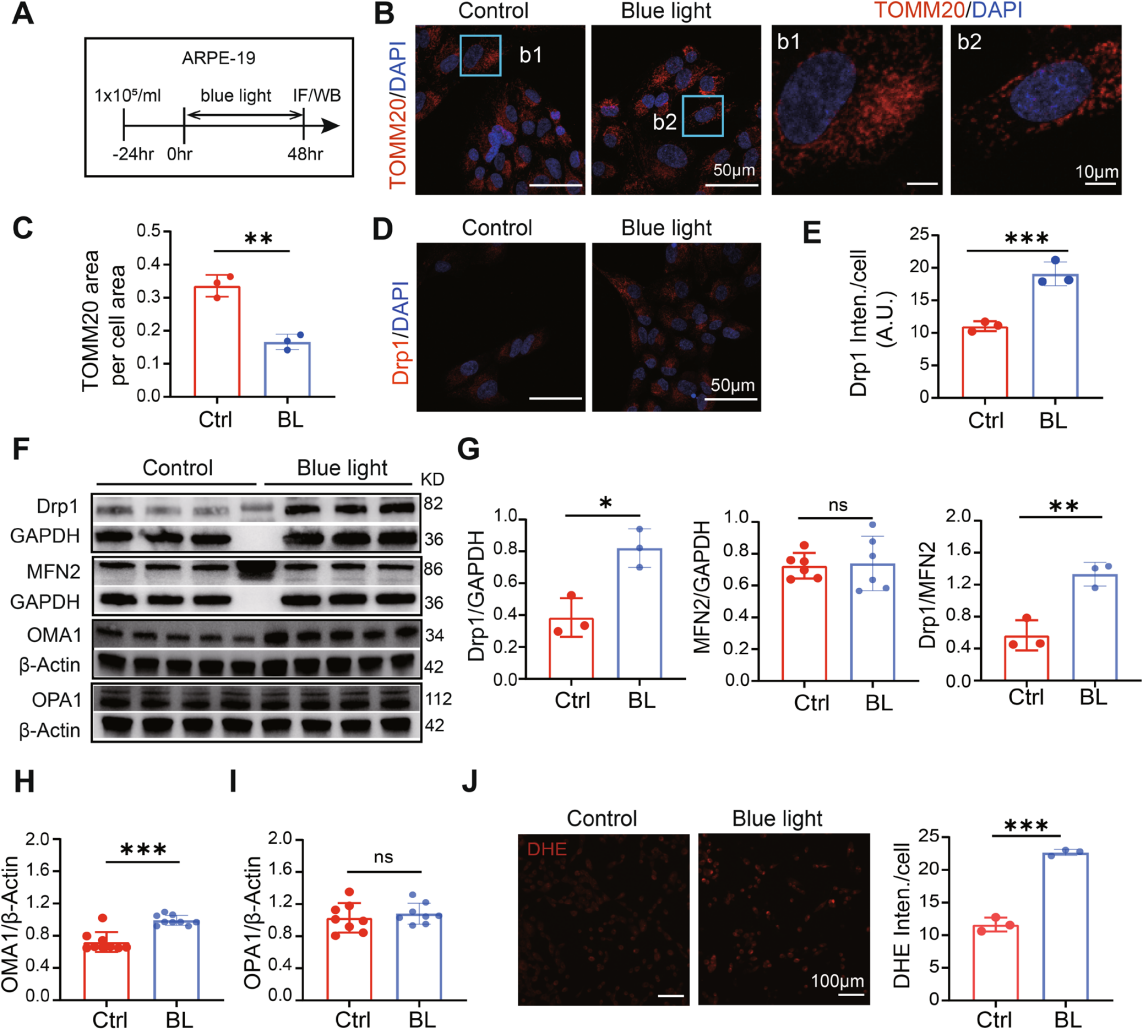
Prolonged blue light exposure induces mitochondrial fragmentation and dysfunction in ARPE‐19 cells. (A) Experiment protocol. (B) Images of ARPE‐19 cells stained with mitochondrial marker TOMM20 (red) and DAPI (blue), with the boxed region enlarged and shown in the bottom panel (b1, b2). TOMM20 staining showed shortened and even fragmented mitochondrial morphology in the ARPE‐19 cell line after 48 h of blue light exposure. (C) Average ratio of TOMM20 fluorescent area in total cell area. (D) Images of ARPE‐19 cells stained with mitochondrial division‐associated protein Drp1 (red) and DAPI (blue). (E) Drp1 intensity per cell. (F) Western Blotting results of the mitochondrial fission proteins Drp1, OMA1, and mitochondrial fusion proteins MFN2, OPA1 from APRE‐19 cells. GAPDH and β‐Actin were used as internal references. (G–I) Quantification of WB results. The expression of Drp and OMA increased, while MFN2 and OPA1 remained unchanged in RPE after blue light exposure. (J) DHE staining in ARPE‐19 cells showed an increase in reactive oxygen species (ROS) level after blue light irradiation. *, *p* < 0.05; **, *p* < 0.01; ***, *p* < 0.001; ns, no significantly different; *t*‐test.

To investigate the effects of blue light on mitochondrial dynamics, we exposed ARPE‐19 cells to blue light (Figure 1A) and assessed mitochondrial morphology using TOMM20 immunofluorescence staining. In the control group, mitochondria appeared long and formed a network around the nuclei stained with DAPI. However, after 48 h of blue light exposure, mitochondria became shortened and fragmented, with a significant reduction in the total TOMM20 fluorescent area per cell, indicating disrupted mitochondrial morphology (Figure 1B,C).

Furthermore, we examined the changes in Drp1, a key protein involved in mitochondrial division. The immunofluorescence results showed a significant increase in Drp1 fluorescent intensity in the blue light‐irradiated group compared to the control group (Figure 1D,E). This increase in Drp1 expression was confirmed by Western blotting (Figure 1F,G left). In contrast, the expression of MFN2, a key protein for mitochondrial fusion, remained unchanged after blue light injury (Figure 1F,G middle). Consequently, the ratio of Drp1 to MFN2 protein expression increased significantly (Figure 1G right), suggesting a shift in mitochondrial kinetic balance to excessive fission. Similar results were observed in another mitochondrial division protein OMA1 (Figure 1F,H) and mitochondrial fusion protein OPA1 (Figure 1F,I).

Additionally, blue light induced mitochondrial dysfunction in ARPE‐19 cells, as evidenced by increased ROS levels detected using DHE staining. The fluorescence intensity of DHE significantly increased after blue light exposure (Figure 1J), indicating oxidative stress in ARPE‐19 cells. In summary, blue light irradiation promotes mitochondrial division and disrupts mitochondrial dynamics in ARPE‐19 cells, potentially contributing to oxidative stress.

### 3.2 Blue Light Induces Epithelial‐Mesenchymal Transition in RPE Cells

**FIGURE 2:**
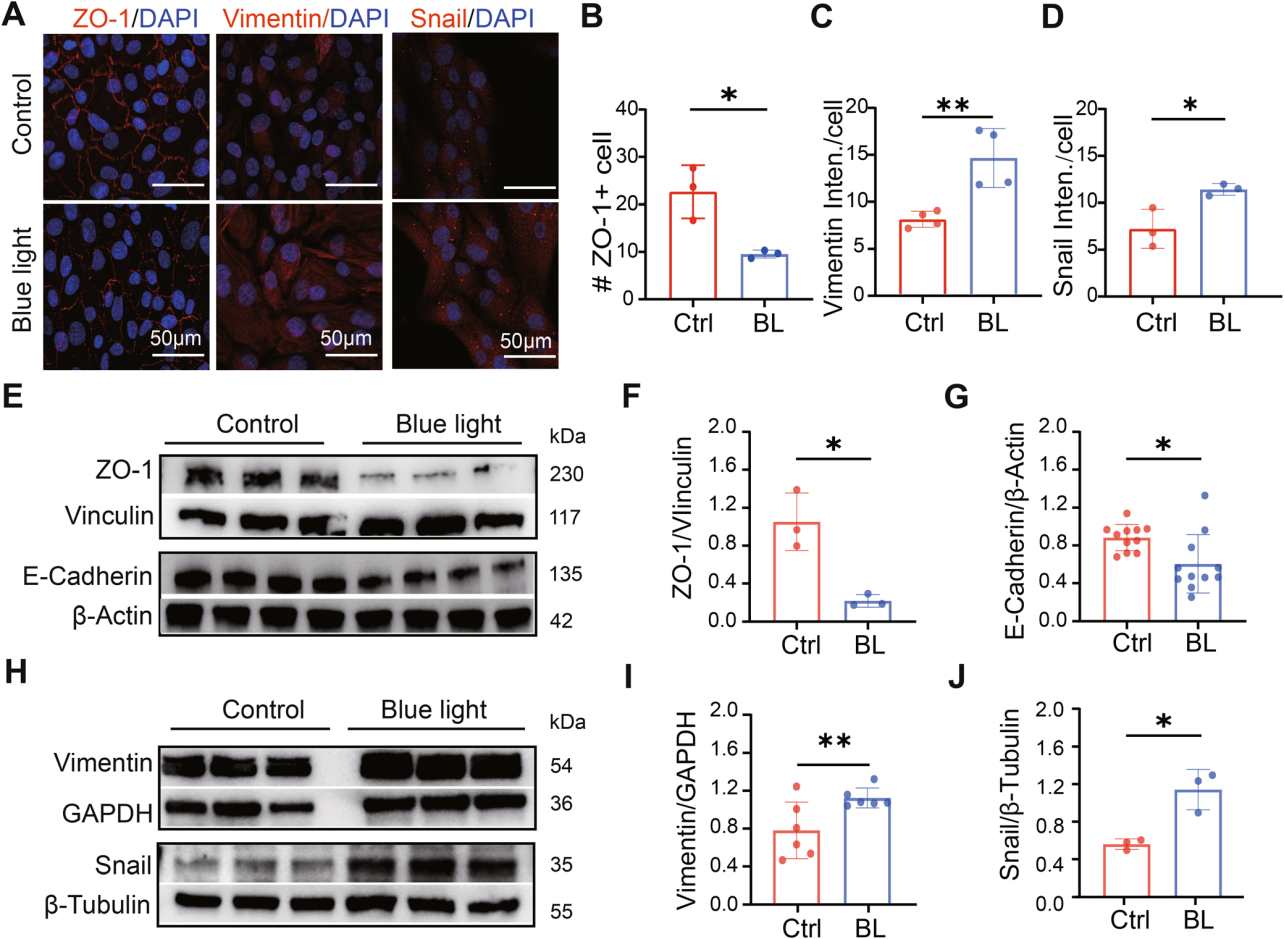
ARPE‐19 undergoes EMT after blue light irradiation. (A) Images of ARPE‐19 cells stained with epithelial cell marker ZO‐1 (left), mesenchymal‐like cell markers Vimentin (middle), and Snail1 (right) in control and blue‐light‐damaged conditions. (B) Number of ZO‐1 positive cells with clear border. (C, D) Average fluorescence intensity of Vimentin (C) and Snail (D) in each cell. (E) Western blotting gels showing expression of the epithelial cell markers ZO‐1 and E‐Cadherin, with Vinculin and β‐Actin as the reference, respectively. (F, G) Statistical results of WB results on ZO‐1 (F), E‐Cadherin (G). (H) Western blotting gel showing expression of mesenchymal cell markers Vimentin and Snail with GAPDH and β‐Tubulin as reference, respectively. (I, J) Statistical results of WB results on Vimentin (I), Snail (J). The expression of the epithelial cell marker ZO‐1 and E‐Cadherin decreased, and the mesenchymal‐like cell markers Vimentin and Snail increased after blue light exposure. *, *p* < 0.05; **, *p* < 0.01; *t*‐test.

To assess whether blue light triggers epithelial‐mesenchymal transition (EMT) in ARPE‐19 cells, we evaluated the expression levels of key EMT markers. Specifically, we compared the epithelial marker ZO‐1 and E‐Cadherin with the mesenchymal markers Vimentin and Snail1. Immunostaining results demonstrated that blue light irradiation reduced ZO‐1 expression while increasing Vimentin and Snail1 expression (Figure 2A–D). Subsequently, the expression of these markers was tested by western blotting (Figure 2E, H). Quantitative analysis of protein expression showed that the expression of epithelial cell markers ZO‐1 and E‐Cadherin was significantly decreased by blue light (Figure 2F, G), while the expression of mesenchymal cell markers Vimentin and Snail1 was significantly increased after blue light irradiation in ARPE‐19 cells (Figure 2I, J). These results suggest that blue light irradiation induces EMT in ARPE‐19 cells, characterized by the loss of epithelial markers and the gain of mesenchymal‐like markers.

### 3.3 Drp1‐Inhibitor Mdivi‐1 Inhibits Excessive Mitochondrial Fission in Blue Light‐Injured RPE Cells

**FIGURE 3:**
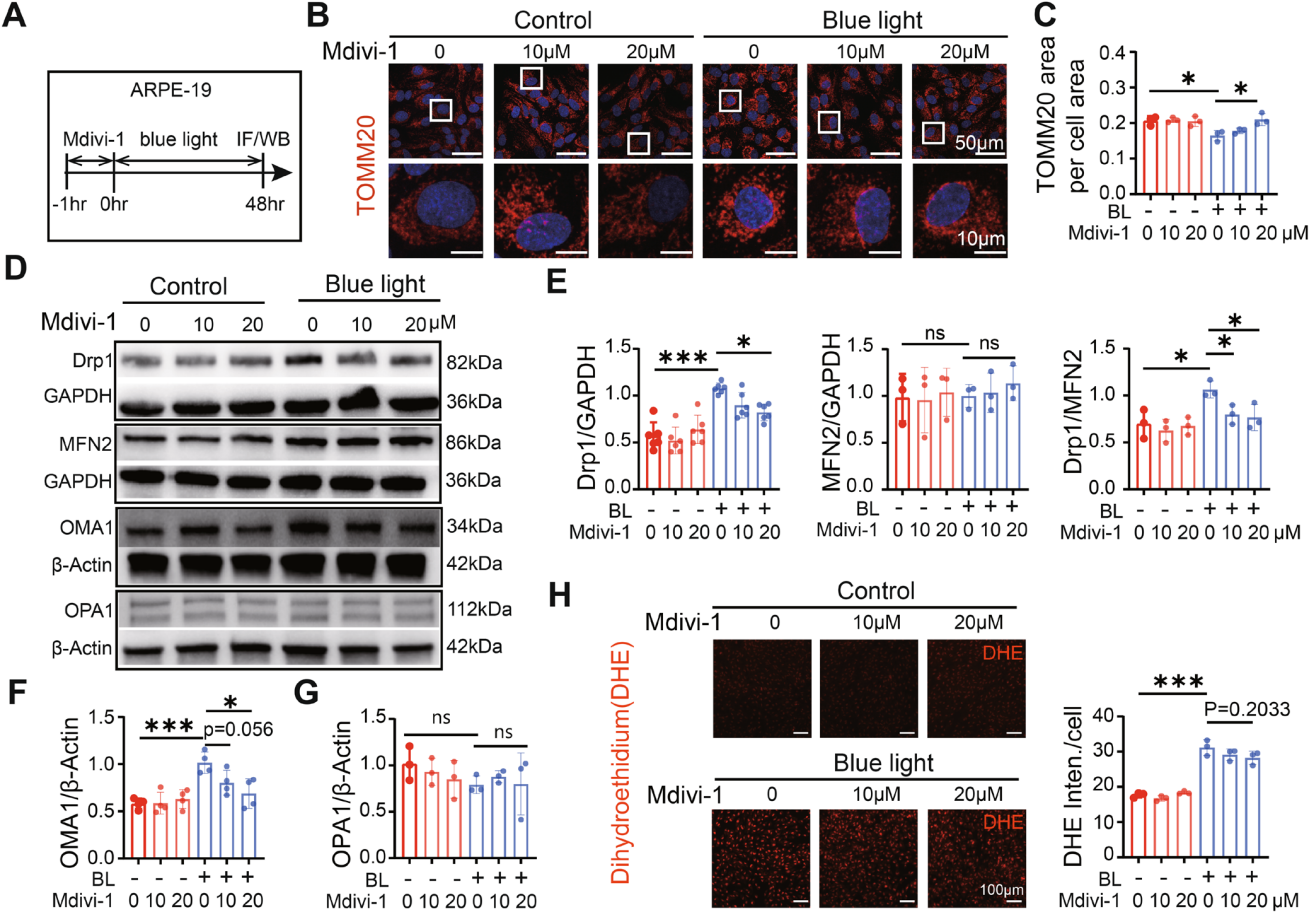
Mdivi‐1 inhibits blue light‐induced mitochondrial division in APRE‐19 cells. (A) Experiment protocol. (B) Images of APRE‐19 stained with mitochondrial structural protein TOMM20 under different Mdiv‐1 dosage treatments. (C) Average ratio of TOMM20 fluorescent area to cell area. Mdivi‐1 treatment restored the TOMM20 level after blue light injury. (D) WB gels of mitochondrial fission proteins Drp1 and OMA1 with mitochondrial fusion proteins MFN2 and OPA1 after blue light irradiation and Mdiv‐1 treatment. (E) Quantitative analysis of WB. Mdivi‐1 treatment restored the ratio of Drp1/MFN2, therefore, the balance of mitochondrial dynamics. (F, G) Western blot quantification. Mdiv‐1 treatment slowed down the blue light‐induced rise in OMA1 (F) expression but hardly affected OPA1 (G), thereby slowing down the excessive mitochondrial fission. (H) DHE staining in ARPE19 cells under different conditions and the quantification of DHE immunofluorescence intensity.*, *p* < 0.05; **, *p* < 0.01; ***, *p* < 0.001; ns, no significantly different; two‐way ANOVA followed by Sidak's post hoc test.

To investigate the relationship between blue light‐induced EMT in ARPE‐19 cells and changes in mitochondrial dynamics, we pretreated the cells with the Drp1‐specific inhibitor Mdivi1 for 1 h before blue light injury to inhibit mitochondrial division (Figure 3A). Two safe concentrations of 10 μM and 20 μM, identified in preliminary experiments (Figure S1A,B), were applied. First, we examined the effect of Mdivi1 on the mitochondrial dynamics balance. Immunofluorescence results revealed that Mdivi1 effectively mitigated blue light‐induced mitochondrial shortening and fragmentation (Figure 3B). Specifically, the average fluorescent area of TOMM20 per cell, a marker of mitochondrial mass, was restored to control levels in a dose‐dependent manner after Mdivi1 treatment (Figure 3C). Western blotting further verified these findings: Mdivi1 pretreatment significantly inhibited the blue light‐induced up‐regulation of the mitochondrial division proteins Drp1 and OMA1, with little effect on the mitochondrial fusion proteins MFN2 and OPA1, thus restoring the mitochondrial dynamic equilibrium (Figure 3D–G). In addition, the fluorescence intensity of DHE in the Mdivi‐1‐treated group showed a decreasing trend compared with that in the blue light‐damaged group (Figure 3H), suggesting that Mdivi‐1 may attenuate blue‐light‐induced oxidative stress, therefore restoring the mitochondrial function.

### 3.4 Inhibition of Mitochondrial Fission Inhibits EMT in Blue Light‐Injured RPE

**FIGURE 4:**
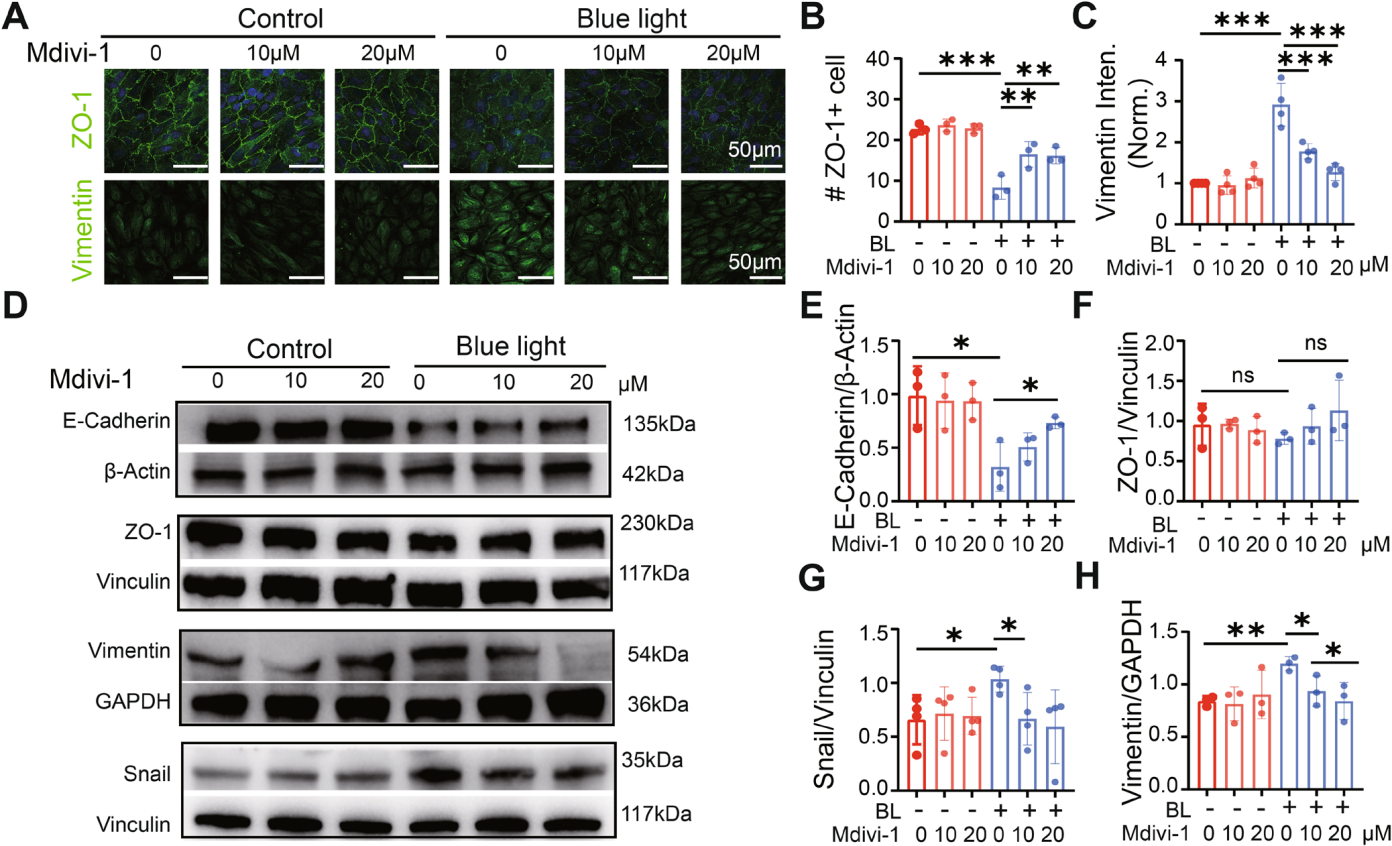
Mdivi1 slows down the blue light‐induced EMT process in ARPE19. (A) Images of APRE‐19 stained with epithelial marker ZO‐1 and mesenchymal marker Vimentin. (B) Number of ZO‐1 positive cells per image. (C) Normalized fluorescence intensity of Vimentin. Mdivi‐1 restored the expression of ZO‐1 and Vimentin in each cell after blue light injury. (D) WB of E‐Cadherin, ZO‐1, Vimentin, and Snail1in response to blue‐light irradiation and Mdiv‐1 treatment, with Vinculin, GAPDH, and β‐Actin as the reference (E–H). Quantitative analysis of WB. Mdivi‐1 treatment slowed down the process of EMT induced by blue light irradiation. *, *p* < 0.05; **, *p* < 0.01; ***, *p* < 0.001; ns, no significantly different; two‐way ANOVA followed by Sidak's post hoc test.

After confirming the inhibition effect of Mdivi‐1 on mitochondrial fission, we next examined whether Mdivi‐1 can ameliorate the blue light‐induced EMT process in ARPE‐19. Immunofluorescence results showed that Mdivi‐1 pretreatment reversed the blue light‐induced changes in EMT markers: it prevented the loss of the epithelial marker ZO‐1 and the increase of the mesenchymal marker Vimentin induced by blue light (Figure 4A–C). Furthermore, Mdivi‐1 pretreatment also slowed down the blue‐light‐induced increase in Snail1 and Vimentin protein expression and the decrease in E‐Cadherin expression, but hardly affected ZO‐1 expression (Figure 4D–H). Our results suggest that inhibiting Drp1, thereby blocking mitochondrial fission, prevents the EMT process induced by blue light in ARPE‐19 cells.

### 3.5 Inhibition of Mitochondrial Fission Slows Down the Blue Light‐Induced EMT Process in RPE In Vivo

**FIGURE 5:**
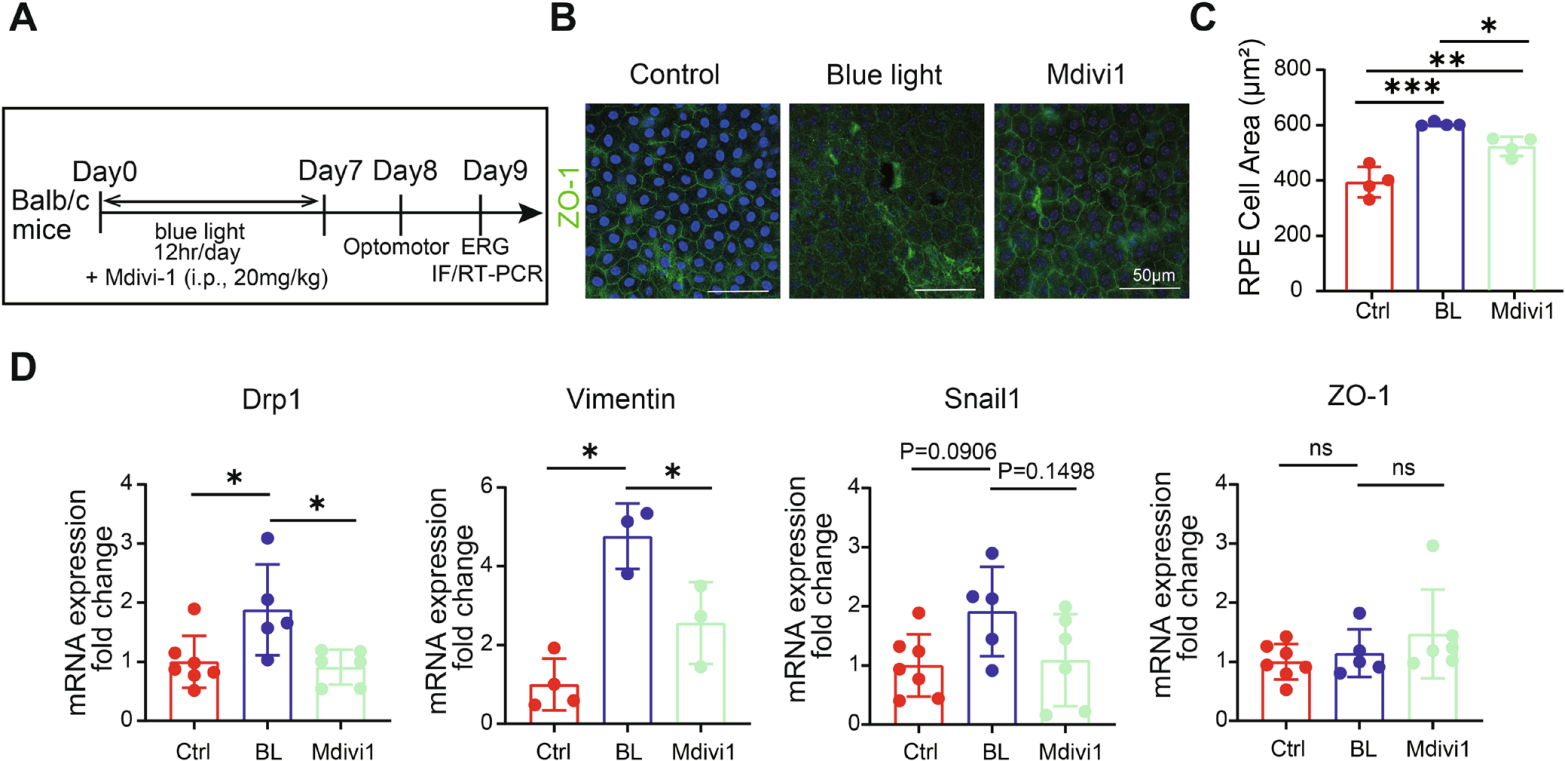
Mdivi‐1 slows down blue light‐induced EMT in mouse RPE. (A) Experiment protocol. (B) Immunofluorescence images of RPE whole mount stained with ZO‐1. (C) Average area of RPE cells. (D) Changes in mRNA expression of EMT markers in RPE‐choroid complex in mice after blue light irradiation and drug treatment. Mdivi‐1 treatment reduced the elevated mRNA expression of Drp1 and Vimentin in mouse RPE caused by blue light exposure.*, *p* < 0.05; **, *p* < 0.01; ***, *p* < 0.001; ns, not significantly different; one‐way ANOVA followed by Turkey's multiple comparison.

Given that inhibiting mitochondrial fission with Mdivi‐1 protected RPE cells against blue light injury in vitro, we sought to determine if Mdivi‐1 could also rescue mouse RPE from blue light induced EMT process. In preliminary experiments, we identified a safe dose of intraperitoneal injection of Mdivi‐1 to be 20 mg/kg body weight (Figure S1C–I). Subsequently, Mdivi‐1 was administered daily to mice during blue light exposure, following the protocol outlined in Figure 5A. In terms of morphology and structure, Mdivi‐1 intervention effectively slowed down the blue light‐induced morphological disruption of RPE cells (Figure 5B,C). Besides, RT‐qPCR results showed that the mRNA expression of mitochondrial fission marker Drp1, as well as mesenchymal cell markers (Vimentin and Snail1) in the RPE‐choroid complex, was significantly increased after blue light irradiation, and Mdivi‐1 treatment effectively slowed down these changes. However, both blue light and Mdivi‐1 had very weak effects on the mRNA expression of the epithelial cell marker ZO‐1(Figure 5D). Our results imply that blue light induces EMT in mouse RPE, and Mdivi‐1 may slow down the blue light‐induced EMT process in RPE in vivo.

### 3.6 Inhibition of Mitochondrial Fission Partially Rescues the Blue Light Impaired Visual Function and the Retinal Cone Structure

**FIGURE 6:**
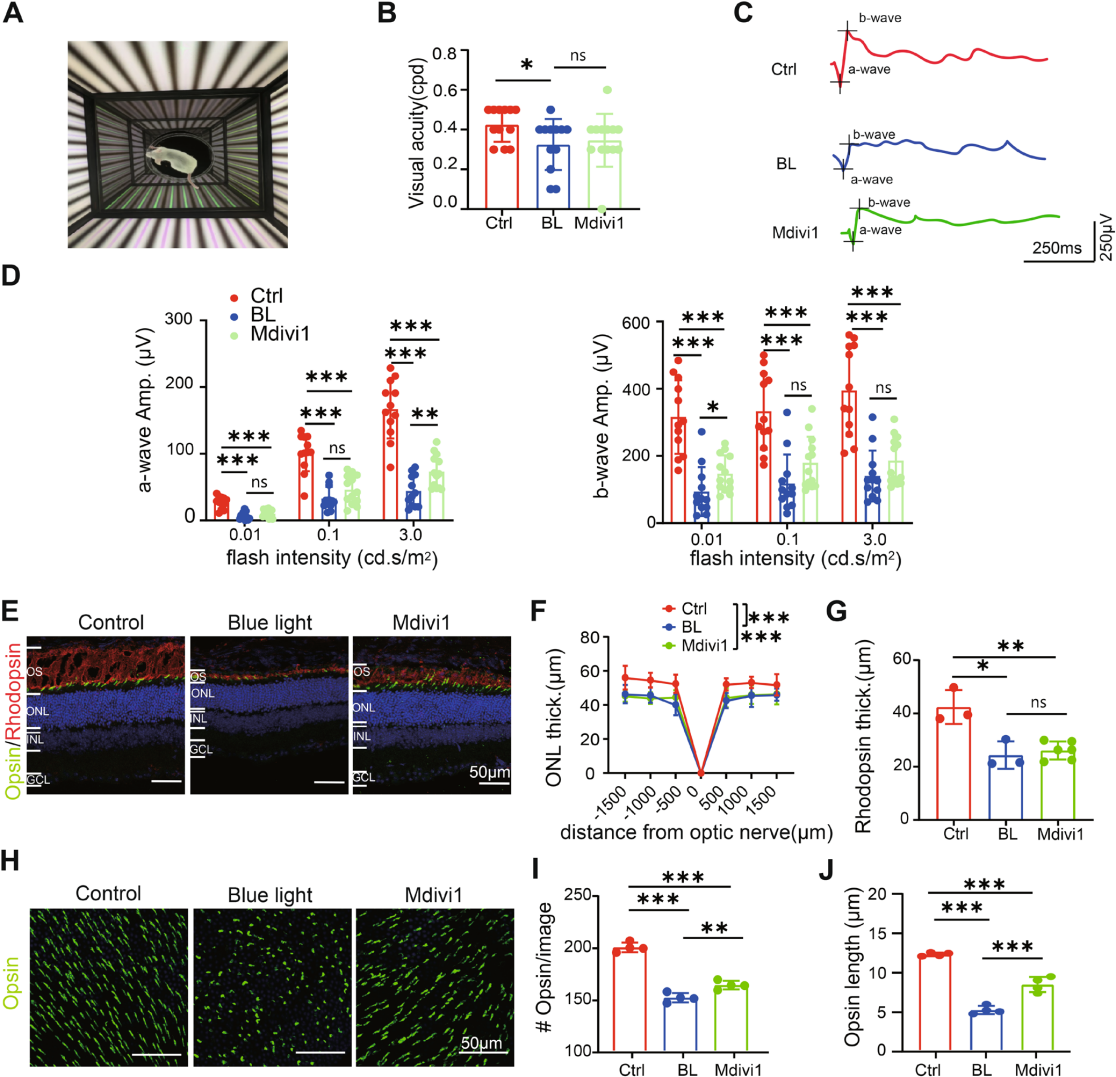
Mdivi1 partially slows the decline of visual function and retinal structure induced by blue light damage. (A) Illustration of the optomotor system that tested the visual acuity. (B) The result of the visual acuity test in control mice (Ctrl), blue light‐injured mice (BL), and Mdivi‐1 treated injured mice. (C) ERG example traces of mice from three groups under dark adaptation with flash intensity of 3.0 cd.s/m^2^. (D) Average amplitude of ERG a‐wave and b‐wave. Mdiv1 improved the amplitude of ERG responses at certain flash intensities. (E) Immunofluorescence images of retinal sections stained with opsin (green), rhodopsin (red), and DAPI (blue). (F) Average thickness of the ONL layer at various distances away from the center of the optic nerve head. (G) Average thickness of rhodopsin layer. (H) Immunofluorescence images of retinal whole‐mount stained with opsin. (I, J) The density and the length of cone outer segment stained by opsin in the whole‐mount retina. Midiv1 improved the structure of cones. *, *p* < 0.05; **, *p* < 0.01; ***, *p* < 0.001; ns, not significantly different; one‐way ANOVA followed by Turkey's multiple comparison. OS, outer segment; ONL, outer nuclei layer; INL, inner nuclei layer; GCL, ganglion cell layer.

After confirming the effect of Mdivi‐1 on RPE in vivo, we continued to explore whether it can rescue the blue light‐induced damage of visual function and retinal structure in mice. In the testing of visual functions, while Mdivi‐1 failed to restore visual acuity in the mice (Figure 6A,B), it did improve the peak amplitude of a‐waves and b‐waves under certain flash intensities (Figure 6C,D). Structurally, while Mdivi‐1 failed to improve the thickness of the outer nuclear layer (ONL) (Figure 6E,F) or the expression of rhodopsin in rods (Figure 6E,G), it significantly improved the density and length of cone outer segment (stained by opsin) on whole‐mount retina as an average of all regions (Figure 6H–J) or different central‐fugal regions (Figure S3). Collectively, these results suggest that inhibiting mitochondrial fission with Mdivi‐1 can partially rescue the blue light impaired visual function and the structure of the retina in mice exposed to blue light. Specifically, Mdivi‐1 appears to protect cone photoreceptors from blue light damage, although its effects on rod photoreceptors are limited.

## 4 Discussion

Excessive light exposure is a known risk factor for AMD (Marquioni‐Ramella and Suburo 2015; Sui et al. 2013). In our study, blue light irradiation in mice and cultured RPE cells led to RPE and photoreceptor damage and decreased visual function, recapitulating AMD‐like features. ARPE‐19 cells exhibited a clear epithelial‐to‐mesenchymal–like transition, consistent with previous reports that light and oxidative stress can trigger RPE EMT (Iriyama et al. 2008; Baek et al. 2017). Blue light also induced excessive mitochondrial fission, disrupting mitochondrial homeostasis and increasing oxidative stress, similar to other reports (Wang et al. 2023). Importantly, we found that inhibiting mitochondrial fission delayed EMT onset and mitigated retinal injury, indicating a causal link between mitochondrial dynamics and EMT progression.

The EMT of RPE cells contributes to the progression of both neovascular and dry AMD by promoting loss of epithelial identity, myofibroblast accumulation, and structural disorganization (Shu et al. 2020; Li et al. 2025; Liu et al. 2023). Multiple signaling pathways—including TGF‐β, bone morphogenetic protein, Wnt, Hedgehog, and receptor tyrosine kinases—have been implicated in regulating RPE EMT (Feng et al. 2024; Mertz et al. 2021; Lee et al. 2007; Zou et al. 2020; Park and Kim 2018). Consistent with previous work showing that blocking EMT alleviates RPE dysfunction (Chen et al. 2025; Han et al. 2023), our study demonstrates that inhibiting EMT in a blue‐light–induced dry AMD model preserved RPE structure and improved retinal function. But unlike those studies mainly focusing on signal pathways involved in the EMT of RPE, we focused on the role of mitochondria, specifically the mitochondrial dynamics.

Mitochondrial dynamics influence diverse cellular processes, and excessive mitochondrial fission has been associated with neurodegeneration, metabolic disorders, and cancer (Sridharan et al. 2024; Iwata et al. 2025; He et al. 2024; Shi et al. 2025). Increased Drp1‐mediated fragmentation (Zeng et al. 2023) and reduced MFN1/2 (Lu et al. 2025; Yao et al. 2025) or OPA1 expression (Iriyama et al. 2008) commonly lead to impaired mitochondrial function and elevated ROS. Pharmacological inhibition of Drp1 using Mdivi‐1 has shown neuroprotective effects in multiple brain and retinal disease models (Kim et al. 2015; Park et al. 2011; Bordt et al. 2022; Fan et al. 2017; Li et al. 2018). Similarly, in our study, blue light induced pronounced mitochondrial fragmentation in ARPE‐19 cells and retinal degeneration in mice, and Mdivi‐1 effectively reduced mitochondrial fission, preserved mitochondrial function, and improved visual responses. These results support a functional role for mitochondrial dynamics in RPE stress responses. Furthermore, we identify the mitochondrial dynamics as the upstream regulator of EMT progression, as Midiv‐1 prevents the EMT progression in RPE.

Although mitochondrial dynamics have been linked to EMT regulation in cancer cells (Ghosh et al. 2023; Wang et al. 2024; Zhang et al. 2023), the mechanisms connecting these processes in RPE cells remain poorly defined. Multiple EMT‐related signaling pathways (such as Notch (Chen et al. 2014), Wnt (Liu et al. 2024), PI3K‐AKT (Chen et al. 2025), TGF‐β (Sreekumar et al. 2025), and NF‐κB (Han et al. 2023)) are sensitive to ROS, mtDNA release, and metabolic stress, all of which can arise from excessive mitochondrial fission. Thus, disrupted mitochondrial dynamics may activate these EMT transcription‐modulated pathways by enhancing oxidative stress or inflammatory signaling. Additionally, altered mitochondrial function may impact calcium buffering and acetyl‐CoA availability (Ying et al. 2025), influencing calcineurin–NFAT pathways or epigenetic regulation (Sample et al. 2023). While our findings indicate that mitochondrial fission contributes to RPE EMT, the specific molecular links require further investigation.

It's worth noting that the expression of OPA1, a critical protein for mitochondrial fusion, did not change with blue light injury or Mdiv‐1 treatment. This suggests that alterations in mitochondrial fusion are likely governed by mechanisms beyond simple protein abundance. Indeed, the critical regulation of mitochondrial fusion by OPA1 is known to occur primarily at the level of post‐translational processing and isoform balance (Gilkerson et al. 2021; Noone et al. 2022), not necessarily total expression, as we measured here. It's possible that stress‐induced cleavage of the long, fusion‐active isoform (L‐OPA1) to short isoforms (S‐OPA1) can inhibit fusion activity without altering total protein abundance. We therefore propose that blue light stress likely impairs fusion through a similar mechanism—altering OPA1 processing or localization—rather than by changing its expression. While our western blot showed two bands for OPA1, it's not certain whether these two bands match L or S‐OPA1, respectively. Further experiments (e.g., isoform‐specific analysis) separating L‐ from S‐OPA1 and calculating the ratio of L‐OPA1/S‐OPA1 may help to verify our proposal.

Besides the unclear mechanism of how mitochondrial dynamics modulates EMT in RPE, there are a few more limitations in our current study. First, only Mdivi‐1 was applied as a specific inhibitor of Drp1 to inhibit mitochondrial fission; it would be beneficial to use other inhibitors of Drp1 or Drp1 siRNA to study the effect of mitochondrial fission on blue‐light induced injury on RPE. For the animal study, the effect of Mdivi‐1 on light‐injured mice is limited; no obvious improvement of RPE structure was observed. Changing the administration way of Mdivi‐1 from i.p. injection to intravitreal injection may be more specific to target the eye; therefore, improve the treatment effect. On the other hand, as intravitreal injection cannot be applied as frequently as daily, injection of Drp1 siRNA by one dose may have advantages over the chemicals, which have to be applied frequently. Second, while our study demonstrated changes in mitochondrial fission (via Drp1, OMA1) and fusion (MFN2, OPA1), it does not fully explore mitochondrial dynamics. To strengthen the mechanistic claims, additional assays should be carried out in a future study to check the autophagy/mitophagy markers, mitochondrial membrane potential markers, and mitochondrial biogenesis markers.

## 5 Conclusion

In conclusion, the present study demonstrates that blue light causes an imbalance of mitochondrial dynamics and the occurrence of EMT in RPE cells in vitro, and impairs retinal function and structure in vivo. Maintaining the balance of mitochondrial dynamics by inhibiting excessive fission slows down the progress of EMT in RPE and retinal impairment. Therefore, our finding demonstrates that Drp1‐mediated excessive mitochondrial fission drives EMT in RPE cells under blue light, which provides new insight into the link between light‐induced mitochondrial dysfunction and RPE EMT. It further suggests that targeting mitochondrial dynamics and EMT may help to treat AMD in the clinic.

## Author Contributions

**Zhi‐Yuan Li** and **Yongxia Huang:** designing research studies. **Zhi‐Yuan Li**, **Dashuang Yang**, **Yintian Li**, and **Yongxia Huang:** conducting experiments. **Zhi‐Yuan Li**, **Yintian Li**, and **Dashuang Yang:** acquiring and analyzing data. **Zhi‐Yuan Li**, **Dashuang Yang**, and **Yintian Li:** writing of original draft. **Ying Xu** and **Tianyun Zhao:** review and editing. **Ying Xu:** supervision and project administration. **Ying Xu** and **Tianyun Zhao:** funding acquisition.

## Funding

This work was supported by the grants from the Natural Science Foundation of Guangdong Province (#2023A1515012397 to Ying Xu), Guangzhou Science and Technology Projects (#2023A03J0899 to Tianyun Zhao).

## Ethics Statement

This study was approved by the Institutional Animal Care and Use Committee of Jinan University (IACUC‐20241018‐06, Guangzhou, China). All operations in the experiment followed the regulations of the NIH and the Experimental Animal Ethics Committee of Jinan University.

## Consent

All authors give their consent for the publication of this manuscript.

## Conflicts of Interest

The authors declare no conflicts of interest.

## Supporting information


**Figure S1:** Pharmacotoxicity testing of Mdivi‐1 in vivo and in vitro. (A) Images of APRE‐19 cells after treatment with different concentrations of Mdivi‐1 for 48 h. (B) Quantification of ARPE‐19 cell activity in different concentrations of Mdivi‐1 using the CCK‐8 kit. Mdivi‐1 at 40 μM is pharmacotoxic to ARPE‐19. (C) The survival rate of mice after i.p injection of different doses of Mdivi‐1. (D) The body weight changes of mice after i.p injection of different doses of Mdivi‐1. The i.p. injection of Mdivi‐1 at a dose of 50 mg/kg was highly toxic to mice, whereas 20 mg/kg and 10 mg/kg had no significant side effect on survival and body weight changes in mice. (E) Immunofluorescence images of retinal sections stained with opsin (red) and DAPI (blue) after intraperitoneal injection of mdivi‐1 at safe concentrations of 10 mg/kg and 20 mg/kg. (F, G) Density and length quantification of mouse retinal opsin. (H, I) Average thickness of the ONL layer at various distances away from the center of the optic nerve (H) and as the mean of the whole retina (I). The i.p. injection of Mdivi‐1 at 20 mg/kg and 10 mg/kg had no side effects on the retina of mice. **, *p* < 0.01; ***, *p* < 0.001; ns, not significantly different; one‐way ANOVA followed by Turkey's multiple comparison. Note that the drug concentrations are reported in μM for in vitro experiments and as administered dose (mg/kg) for in vivo experiments, consistent with field standards.

**Figure S2:** Mdivi‐1 slows blue light‐induced retinal cone degeneration at various central‐fugal regions. (A) Schematic of the chosen regions across various central‐fugal distances in the experiment. (B) Immunofluorescence images of retinal whole‐mount stained with opsin at various regions. (C, D) The density and length of opsin in the cone outer segment in various regions on whole‐mount retina. **, *p* < 0.01; ***, *p* < 0.001; ns, not significantly different; one‐way ANOVA followed by Turkey's multiple comparison.

**Appendix S1:** acel70416‐sup‐0003‐AppendixS1.pdf.

## Data Availability

The data that support the findings of this study are available on request from the corresponding author.

## References

[acel70416-bib-0001] Alaimo, A. , G. G. Liñares , J. M. Bujjamer , et al. 2019. “Toxicity of Blue Led Light and A2E Is Associated to Mitochondrial Dynamics Impairment in ARPE‐19 Cells: Implications for Age‐Related Macular Degeneration.” Archives of Toxicology 93, no. 5: 1401–1415. 30778631 10.1007/s00204-019-02409-6

[acel70416-bib-0002] Ashraf, R. , and S. Kumar . 2022. “Mfn2‐Mediated Mitochondrial Fusion Promotes Autophagy and Suppresses Ovarian Cancer Progression by Reducing ROS Through AMPK/mTOR/ERK Signaling.” Cellular and Molecular Life Sciences 79, no. 11: 573. 36308626 10.1007/s00018-022-04595-6 PMC11803038

[acel70416-bib-0003] Baek, A. , S. Yoon , J. Kim , et al. 2017. “Autophagy and KRT8/Keratin 8 Protect Degeneration of Retinal Pigment Epithelium Under Oxidative Stress.” Autophagy 13, no. 2: 248–263. 28045574 10.1080/15548627.2016.1256932 PMC5324842

[acel70416-bib-0004] Blasiak, J. , P. Sobczuk , E. Pawlowska , and K. Kaarniranta . 2022. “Interplay Between Aging and Other Factors of the Pathogenesis of Age‐Related Macular Degeneration.” Ageing Research Reviews 81: 101735. 36113764 10.1016/j.arr.2022.101735

[acel70416-bib-0005] Bordt, E. A. , N. Zhang , J. Waddell , and B. M. Polster . 2022. “The Non‐Specific Drp1 Inhibitor Mdivi‐1 Has Modest Biochemical Antioxidant Activity.” Antioxidants (Basel) 11, no. 3: 450. 35326100 10.3390/antiox11030450 PMC8944504

[acel70416-bib-0006] Bresciani, G. , F. Manai , S. Felszeghy , A. Smedowski , K. Kaarniranta , and M. Amadio . 2024. “VEGF and ELAVL1/HuR Protein Levels Are Increased in Dry and Wet AMD Patients. A New Tile in the Pathophysiologic Mechanisms Underlying RPE Degeneration?” Pharmacological Research 208: 107380. 39216841 10.1016/j.phrs.2024.107380

[acel70416-bib-0007] Buonfiglio, F. , C. A. Korb , B. Stoffelns , N. Pfeiffer , and A. Gericke . 2024. “Recent Advances in Our Understanding of Age‐Related Macular Degeneration: Mitochondrial Dysfunction, Redox Signaling, and the Complement System.” Aging and Disease 16, no. 3: 1535–1575. 38421830 10.14336/AD.2024.0124 PMC12096954

[acel70416-bib-0008] Chen, X. , W. Xiao , W. Wang , L. Luo , S. Ye , and Y. Liu . 2014. “The Complex Interplay Between ERK1/2, TGFβ/Smad, and Jagged/Notch Signaling Pathways in the Regulation of Epithelial‐Mesenchymal Transition in Retinal Pigment Epithelium Cells.” PLoS One 9, no. 5: e96365. 24788939 10.1371/journal.pone.0096365 PMC4008562

[acel70416-bib-0009] Chen, Y. , M. Jiang , L. Li , et al. 2025. “Absent in Melanoma 2: A Potent Suppressor of Retinal Pigment Epithelial‐Mesenchymal Transition and Experimental Proliferative Vitreoretinopathy.” Cell Death & Disease 16, no. 1: 49. 39870644 10.1038/s41419-025-07367-9 PMC11772762

[acel70416-bib-0010] Chowdhury, O. , S. Bammidi , P. Gautam , et al. 2025. “Activated mTOR Signaling in the RPE Drives EMT, Autophagy, and Metabolic Disruption, Resulting in AMD‐Like Pathology in Mice.” Aging Cell 24, no. 6: e70018. 39957408 10.1111/acel.70018 PMC12151893

[acel70416-bib-0011] Fan, L. F. , L. Fan , P. He , et al. 2017. “Mdivi‐1 Ameliorates Early Brain Injury After Subarachnoid Hemorrhage via the Suppression of Inflammation‐Related Blood‐Brain Barrier Disruption and Endoplasmic Reticulum Stress‐Based Apoptosis.” Free Radical Biology & Medicine 112: 336–349. 28790012 10.1016/j.freeradbiomed.2017.08.003

[acel70416-bib-0012] Feng, J. , X. Huang , Y. Peng , et al. 2025. “Pyruvate Kinase M2 Modulates Mitochondrial Dynamics and EMT in Alveolar Epithelial Cells During Sepsis‐Associated Pulmonary Fibrosis.” Journal of Translational Medicine 23, no. 1: 205. 39972351 10.1186/s12967-025-06199-7 PMC11837412

[acel70416-bib-0013] Feng, Y. , F. Yang , J. Zheng , et al. 2024. “Circular RNA HIPK3 Mediates Epithelial‐Mesenchymal Transition of Retinal Pigment Epithelial Cells by Sponging Multiple microRNAs.” Scientific Reports 14, no. 1: 28872. 39572643 10.1038/s41598-024-71119-6 PMC11582593

[acel70416-bib-0014] Fleckenstein, M. , T. D. L. Keenan , R. H. Guymer , et al. 2021. “Age‐Related Macular Degeneration.” Nature Reviews. Disease Primers 7, no. 1: 31. 10.1038/s41572-021-00265-2 PMC12878645 33958600

[acel70416-bib-0015] Gao, F. , L. Wang , B. Wu , et al. 2022. “Elimination of Senescent Cells Inhibits Epithelial‐Mesenchymal Transition of Retinal Pigment Epithelial Cells.” Experimental Eye Research 223: 109207. 35926646 10.1016/j.exer.2022.109207

[acel70416-bib-0016] Ghosh, D. , S. Pakhira , D. D. Ghosh , S. Roychoudhury , and S. S. Roy . 2023. “Ets1 Facilitates EMT/Invasion Through Drp1‐Mediated Mitochondrial Fragmentation in Ovarian Cancer.” iScience 26, no. 9: 107537. 37664613 10.1016/j.isci.2023.107537 PMC10469980

[acel70416-bib-0017] Ghosh, S. , P. Shang , H. Terasaki , et al. 2018. “A Role for βA3/A1‐Crystallin in Type 2 EMT of RPE Cells Occurring in Dry Age‐Related Macular Degeneration.” Investigative Ophthalmology & Visual Science 59, no. 4: AMD104–AMD113. 30098172 10.1167/iovs.18-24132 PMC6058694

[acel70416-bib-0018] Gilkerson, R. , P. de la Torre , and S. St. Vallier . 2021. “Mitochondrial OMA1 and OPA1 as Gatekeepers of Organellar Structure/Function and Cellular Stress Response.” Frontiers in Cell and Developmental Biology 9: 9. 10.3389/fcell.2021.626117 PMC8027119 33842456

[acel70416-bib-0019] Han, H. , Y. Yang , Z. Han , et al. 2023. “NFκB‐Mediated Expression of Phosphoinositide 3‐Kinase δ Is Critical for Mesenchymal Transition in Retinal Pigment Epithelial Cells.” Cells 12, no. 2: 207. 10.3390/cells12020207. 36672142 PMC9857235

[acel70416-bib-0020] Hanus, J. , C. Anderson , and S. Wang . 2015. “RPE Necroptosis in Response to Oxidative Stress and in AMD.” Ageing Research Reviews 24: 286–298. 26369358 10.1016/j.arr.2015.09.002 PMC4661094

[acel70416-bib-0021] He, K. , Y. Li , W. Xiong , et al. 2024. “Sevoflurane Exposure Accelerates the Onset of Cognitive Impairment via Promoting p‐Drp1(S616)‐Mediated Mitochondrial Fission in a Mouse Model of Alzheimer's Disease.” Free Radical Biology & Medicine 225: 699–710. 39490772 10.1016/j.freeradbiomed.2024.10.301

[acel70416-bib-0022] Hui, Q. Y. , Q. Hui , N. Yang , et al. 2024. “Isorhamnetin Suppresses the Epithelial‐Mesenchymal Transition of the Retinal Pigment Epithelium Both In Vivo and In Vitro Through Nrf2‐Dependent AKT/GSK‐3β Pathway.” Experimental Eye Research 240: 109823. 10.1016/j.exer.2024.109823. 38331017

[acel70416-bib-0023] Iriyama, A. , T. Iriyama , Y. Tamaki , and Y. Yanagi . 2008. “Effects of White Light on Beta‐Catenin Signaling Pathway in Retinal Pigment Epithelium.” Biochemical and Biophysical Research Communications 375, no. 1: 173–177. 18692477 10.1016/j.bbrc.2008.07.158

[acel70416-bib-0024] Iwata, W. , N. Haggerty , H. Sesaki , and M. Iijima . 2025. “Targeting Mitochondrial Structure and Dynamics for Therapeutic Intervention in Cancer.” PLoS Biology 23, no. 10: e3003453. 41124166 10.1371/journal.pbio.3003453 PMC12543139

[acel70416-bib-0025] Kang, C. 2023. “Avacincaptad Pegol: First Approval.” Drugs 83, no. 15: 1447–1453. 37814173 10.1007/s40265-023-01948-8

[acel70416-bib-0026] Kim, K. Y. , G. A. Perkins , M. S. Shim , et al. 2015. “DRP1 Inhibition Rescues Retinal Ganglion Cells and Their Axons by Preserving Mitochondrial Integrity in a Mouse Model of Glaucoma.” Cell Death & Disease 6, no. 8: e1839. 26247724 10.1038/cddis.2015.180 PMC4558491

[acel70416-bib-0027] Lee, H. , S. J. O'Meara , C. O'Brien , and R. Kane . 2007. “The Role of Gremlin, a BMP Antagonist, and Epithelial‐To‐Mesenchymal Transition in Proliferative Vitreoretinopathy.” Investigative Ophthalmology & Visual Science 48, no. 9: 4291–4299. 17724219 10.1167/iovs.07-0086

[acel70416-bib-0028] Li, D. , Q. Ou , F. Gao , et al. 2025. “CRX Is an Intrinsic Suppressor of Epithelial–Mesenchymal Transition in Retinal Pigment Epithelial Cells: A Promising Therapeutic Avenue for Subretinal Fibrosis.” Cell Death & Disease 17, no. 1: 156. 10.1038/s41419-025-08352-y. 41469516 PMC12859066

[acel70416-bib-0029] Li, J. Y. , K. Zhang , D. Xu , et al. 2018. “Mitochondria! Fission Is Required for Blue Light‐Induced Apoptosis and Mitophagy in Retinal Neuronal R28 Cells.” Frontiers in Molecular Neuroscience 11: 432. 30538621 10.3389/fnmol.2018.00432 PMC6277708

[acel70416-bib-0030] Liu, D. , J. du , H. Xie , et al. 2024. “Wnt5a/β‐Catenin‐Mediated Epithelial‐Mesenchymal Transition: A Key Driver of Subretinal Fibrosis in Neovascular Age‐Related Macular Degeneration.” Journal of Neuroinflammation 21, no. 1: 75. 38532410 10.1186/s12974-024-03068-w PMC10967154

[acel70416-bib-0031] Liu, D. , C. Zhang , J. Zhang , and G.‐T. Xu . 2023. “Molecular Pathogenesis of Subretinal Fibrosis in Neovascular AMD Focusing on Epithelial‐Mesenchymal Transformation of Retinal Pigment Epithelium.” Neurobiology of Disease 185: 106250. 37536385 10.1016/j.nbd.2023.106250

[acel70416-bib-0032] Liu, X. B. , F. Liu , Y. Y. Liang , et al. 2021. “Luteolin Delays Photoreceptor Degeneration in a Mouse Model of Retinitis Pigmentosa.” Neural Regeneration Research 16, no. 10: 2109–2120. 33642401 10.4103/1673-5374.303537 PMC8343326

[acel70416-bib-0033] Livak, K. J. , and T. D. Schmittgen . 2001. “Analysis of Relative Gene Expression Data Using Real‐Time Quantitative PCR and the 2(‐Delta Delta C(T)) Method.” Methods 25, no. 4: 402–408. 11846609 10.1006/meth.2001.1262

[acel70416-bib-0034] Lu, D. , Y. Yang , G. Huang , et al. 2025. “MFN2 and BAG6 Synergistically Protect Against Cerebral Reperfusion Injury by Regulating ROS Levels and Autophagic Flux.” Stroke 56, no. 12: 3468–3483. 40891328 10.1161/STROKEAHA.125.052689 PMC12643567

[acel70416-bib-0035] Lugassy, Y. , E. Berent , L. Tarony , et al. 2025. “HDAC Inhibition Protects RPE Cells From Oxidative Stress via Enhanced Mitochondrial Fusion, Cytoskeletal Repair, and Nrf‐2 Activation.” Free Radical Biology & Medicine 240: 59–70. 40816649 10.1016/j.freeradbiomed.2025.08.007

[acel70416-bib-0036] Marquioni‐Ramella, M. D. , and A. M. Suburo . 2015. “Photo‐Damage, Photo‐Protection and Age‐Related Macular Degeneration.” Photochemical & Photobiological Sciences 14, no. 9: 1560–1577. 26198091 10.1039/c5pp00188a

[acel70416-bib-0037] Mertz, J. L. , S. R. Sripathi , X. Yang , et al. 2021. “Proteomic and Phosphoproteomic Analyses Identify Liver‐Related Signaling in Retinal Pigment Epithelial Cells During EMT.” Cell Reports 37, no. 3: 109866. 34686321 10.1016/j.celrep.2021.109866

[acel70416-bib-0038] Noone, J. , D. J. O'Gorman , and H. C. Kenny . 2022. “OPA1 Regulation of Mitochondrial Dynamics in Skeletal and Cardiac Muscle.” Trends in Endocrinology and Metabolism 33, no. 10: 710–721. 35945104 10.1016/j.tem.2022.07.003

[acel70416-bib-0039] Park, G. B. , and D. Kim . 2018. “Cigarette Smoke‐Induced EGFR Activation Promotes Epithelial Mesenchymal Migration of Human Retinal Pigment Epithelial Cells Through Regulation of the FAK‐Mediated Syk/Src Pathway.” Molecular Medicine Reports 17, no. 3: 3563–3574. 29286114 10.3892/mmr.2017.8355 PMC5802154

[acel70416-bib-0040] Park, S. W. , K. Y. Kim , J. D. Lindsey , et al. 2011. “A Selective Inhibitor of drp1, Mdivi‐1, Increases Retinal Ganglion Cell Survival in Acute Ischemic Mouse Retina.” Investigative Ophthalmology & Visual Science 52, no. 5: 2837–2843. 21372007 10.1167/iovs.09-5010 PMC3088566

[acel70416-bib-0041] Sample, R. A. , M. F. Nogueira , R. D. Mitra , and S. V. Puram . 2023. “Epigenetic Regulation of Hybrid Epithelial‐Mesenchymal Cell States in Cancer.” Oncogene 42, no. 29: 2237–2248. 37344626 10.1038/s41388-023-02749-9 PMC10578205

[acel70416-bib-0042] Shen, G. , Y. Li , Y. Zeng , et al. 2023. “Kallistatin Deficiency Induces the Oxidative Stress‐Related Epithelial‐Mesenchymal Transition of Retinal Pigment Epithelial Cells: A Novel Protagonist in Age‐Related Macular Degeneration.” Investigative Ophthalmology & Visual Science 64, no. 12: 15. 10.1167/iovs.64.12.15 PMC10500364 37682567

[acel70416-bib-0043] Shen, M. , A. Berni , J. Liu , et al. 2026. “Real‐World Experience With Intravitreal Pegcetacoplan for the Treatment of Geographic Atrophy in Age‐Related Macular Degeneration.” American Journal of Ophthalmology 281: 31–41. 40915520 10.1016/j.ajo.2025.09.006

[acel70416-bib-0044] Shi, Q. , M. Wu , J. Zhong , et al. 2025. “Procyanidin Capsules Combat ALF by Restoring Mitochondrial Homeostasis and Inhibiting Necroptosis via the PGAM5/DRP1/PINK1 Pathway.” Advanced Science 13: e08742. 41340222 10.1002/advs.202508742 PMC12866713

[acel70416-bib-0045] Shu, D. Y. , E. Butcher , and M. Saint‐Geniez . 2020. “EMT and EndMT: Emerging Roles in Age‐Related Macular Degeneration.” International Journal of Molecular Sciences 21, no. 12: 4271. 32560057 10.3390/ijms21124271 PMC7349630

[acel70416-bib-0046] Sreekumar, P. G. , M. H. Nam , E. Hong , R. Kannan , and R. H. Nagaraj . 2025. “RAGE Is Essential for Subretinal Fibrosis in Laser‐Induced Choroidal Neovascularization: Therapeutic Implications.” Investigative Ophthalmology & Visual Science 66, no. 6: 30. 10.1167/iovs.66.6.30 PMC12161369 40488713

[acel70416-bib-0047] Sridharan, P. S. , Y. Koh , E. Miller , et al. 2024. “Acutely Blocking Excessive Mitochondrial Fission Prevents Chronic Neurodegeneration After Traumatic Brain Injury.” Cell Reports Medicine 5, no. 9: 101715. 39241772 10.1016/j.xcrm.2024.101715 PMC11525032

[acel70416-bib-0048] Sui, G. Y. , G. C. Liu , G. Y. Liu , et al. 2013. “Is Sunlight Exposure a Risk Factor for Age‐Related Macular Degeneration? A Systematic Review and Meta‐Analysis.” British Journal of Ophthalmology 97, no. 4: 389–394. 23143904 10.1136/bjophthalmol-2012-302281

[acel70416-bib-0049] Tan, L. X. , C. J. Germer , N. La Cunza , and A. Lakkaraju . 2020. “Complement Activation, Lipid Metabolism, and Mitochondrial Injury: Converging Pathways in Age‐Related Macular Degeneration.” Redox Biology 37: 101781. 33162377 10.1016/j.redox.2020.101781 PMC7767764

[acel70416-bib-0050] Trivizki, O. , C. C. Wykoff , M. A. Smoor , et al. 2025. “Effect of Pegcetacoplan on Aqueous Humor Proteome in Geographic Atrophy: A Prospective Exploration.” Investigative Ophthalmology & Visual Science 66, no. 15: 24. 10.1167/iovs.66.15.24 PMC12700176 41347874

[acel70416-bib-0051] Wang, L. , X. Yu , D. Zhang , et al. 2023. “Long‐Term Blue Light Exposure Impairs Mitochondrial Dynamics in the Retina in Light‐Induced Retinal Degeneration In Vivo and In Vitro.” Journal of Photochemistry and Photobiology. B, Biology 240: 112654. 36724628 10.1016/j.jphotobiol.2023.112654

[acel70416-bib-0052] Wang, N. , Y. Wang , L. Zhang , W. Yang , and S. Fu . 2025. “Molecular Mechanisms of Epithelial‐Mesenchymal Transition in Retinal Pigment Epithelial Cells: Implications for Age‐Related Macular Degeneration (AMD) Progression.” Biomolecules 15, no. 6: 771. 40563412 10.3390/biom15060771 PMC12191313

[acel70416-bib-0053] Wang, Z. , S. Tang , L. Cai , et al. 2024. “DRP1 Inhibition‐Mediated Mitochondrial Elongation Abolishes Cancer Stemness, Enhances Glutaminolysis, and Drives Ferroptosis in Oral Squamous Cell Carcinoma.” British Journal of Cancer 130, no. 11: 1744–1757. 38582810 10.1038/s41416-024-02670-2 PMC11130175

[acel70416-bib-0054] Yang, Y. C. , Y. Chien , A. A. Yarmishyn , et al. 2024. “Inhibition of Oxidative Stress‐Induced Epithelial‐Mesenchymal Transition in Retinal Pigment Epithelial Cells of Age‐Related Macular Degeneration Model by Suppressing ERK Activation.” Journal of Advanced Research 60: 141–157. 37328058 10.1016/j.jare.2023.06.004 PMC11156608

[acel70416-bib-0055] Yao, Z. , X. Tan , H. Cui , et al. 2025. “NDUFAB1 Promotes Hepatocellular Carcinoma Progression by Disrupting Mitochondrial Homeostasis and Mitophagy Pathway.” International Immunopharmacology 167: 115664. 41106117 10.1016/j.intimp.2025.115664

[acel70416-bib-0056] Ying, Z. , Y. Xin , Z. Liu , et al. 2025. “The Mitochondrial Unfolded Protein Response Inhibits Pluripotency Acquisition and Mesenchymal‐To‐Epithelial Transition in Somatic Cell Reprogramming.” Nature Metabolism 7, no. 5: 940–951. 10.1038/s42255-025-01261-6 40205158

[acel70416-bib-0057] Yu, B. , J. Ma , J. Li , D. Wang , Z. Wang , and S. Wang . 2020. “Mitochondrial Phosphatase PGAM5 Modulates Cellular Senescence by Regulating Mitochondrial Dynamics.” Nature Communications 11, no. 1: 2549. 10.1038/s41467-020-16312-7 PMC7242393 32439975

[acel70416-bib-0058] Zeng, Z. , M. You , C. Fan , R. Rong , H. Li , and X. Xia . 2023. “Pathologically High Intraocular Pressure Induces Mitochondrial Dysfunction Through Drp1 and Leads to Retinal Ganglion Cell PANoptosis in Glaucoma.” Redox Biology 62: 102687. 36989574 10.1016/j.redox.2023.102687 PMC10074988

[acel70416-bib-0059] Zhang, L. , L. Sun , L. Wang , et al. 2023. “Mitochondrial Division Inhibitor (Mdivi‐1) Inhibits Proliferation and Epithelial‐Mesenchymal Transition via the NF‐κB Pathway in Thyroid Cancer Cells.” Toxicology In Vitro 88: 105552. 36621616 10.1016/j.tiv.2023.105552

[acel70416-bib-0060] Zhao, S. , X. Zhang , Y. Shi , et al. 2020. “MIEF2 Over‐Expression Promotes Tumor Growth and Metastasis Through Reprogramming of Glucose Metabolism in Ovarian Cancer.” Journal of Experimental & Clinical Cancer Research 39, no. 1: 286. 33317572 10.1186/s13046-020-01802-9 PMC7737286

[acel70416-bib-0061] Zou, H. , C. Shan , L. Ma , J. Liu , N. Yang , and J. Zhao . 2020. “Polarity and Epithelial‐Mesenchymal Transition of Retinal Pigment Epithelial Cells in Proliferative Vitreoretinopathy.” PeerJ 8: e10136. 33150072 10.7717/peerj.10136 PMC7583629

