# Supplementary figures and images for: Dynamin‐Related Protein 1‐Dependent Disruption of Mitochondrial Homeostasis Drives Blue Light‐Induced Epithelial‐Mesenchymal Transition in Retinal Aging
Source: Aging Cell. 2026 Feb 16;25(2):e70416. doi: 10.1111/acel.70416 (PMC12910175; doi:10.1111/acel.70416)

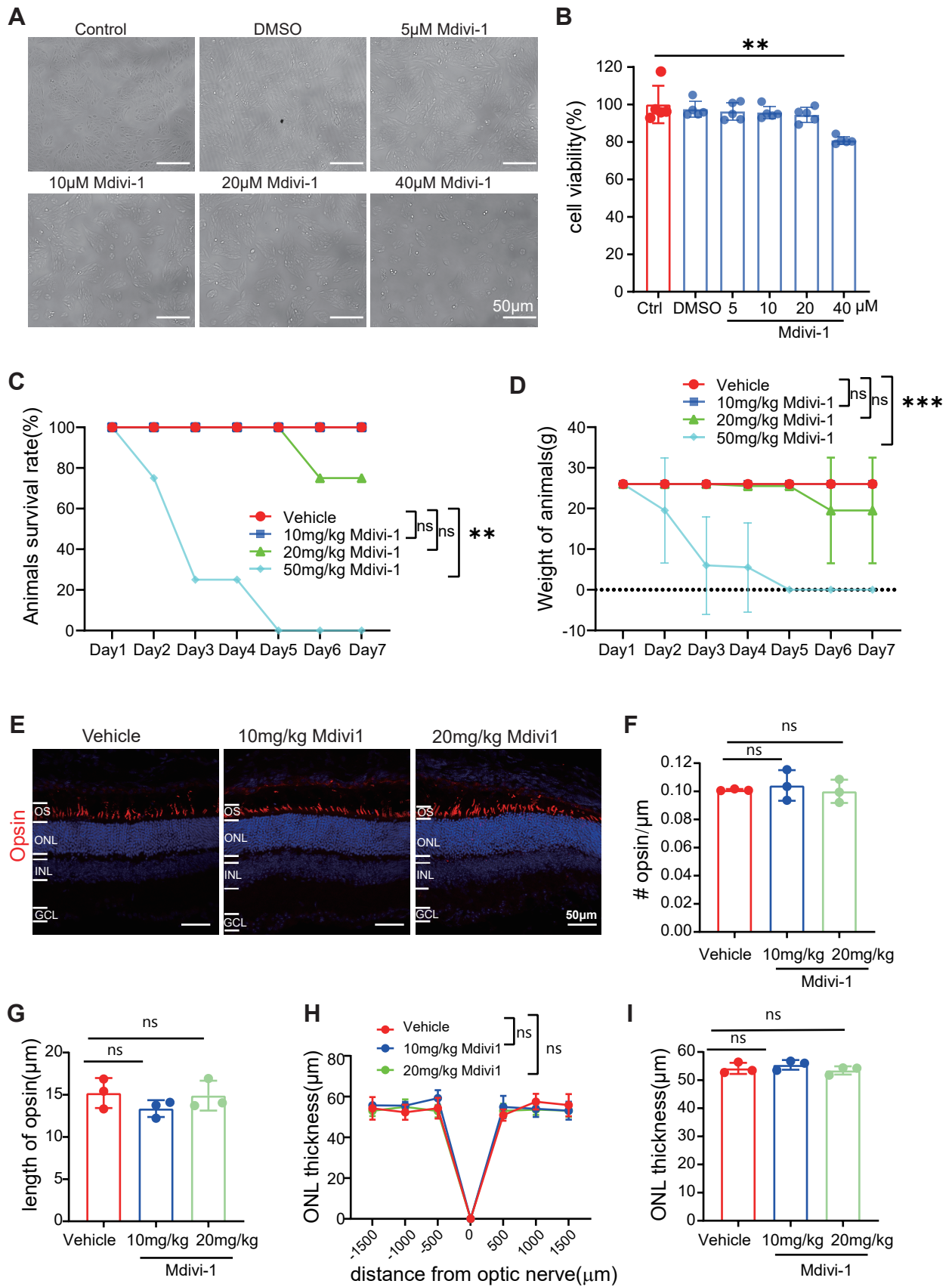

**Supplemental Figure 1**

Supplement: Supplementary file 1 — Figure S1: Pharmacotoxicity testing of Mdivi‐1 in vivo and in vitro. (A) Images of APRE‐19 cells after treatment with different concentrations of Mdivi‐1 for 48 h. (B) Quantification of ARPE‐19 cell activity in different concentrations of Mdivi‐1 using the CCK‐8 kit. Mdivi‐1 at 40 μM is pharmacotoxic to ARPE‐19. (C) The survival rate of mice after i.p injection of different doses of Mdivi‐1. (D) The body weight changes of mice after i.p injection of different doses of Mdivi‐1. The i.p. injection of Mdivi‐1 at a dose of 50 mg/kg was highly toxic to mice, whereas 20 mg/kg and 10 mg/kg had no significant side effect on survival and body weight changes in mice. (E) Immunofluorescence images of retinal sections stained with opsin (red) and DAPI (blue) after intraperitoneal injection of mdivi‐1 at safe concentrations of 10 mg/kg and 20 mg/kg. (F, G) Density and length quantification of mouse retinal opsin. (H, I) Average thickness of the ONL layer at various distances away from the center of the optic nerve (H) and as the mean of the whole retina (I). The i.p. injection of Mdivi‐1 at 20 mg/kg and 10 mg/kg had no side effects on the retina of mice. **, p < 0.01; ***, p < 0.001; ns, not significantly different; one‐way ANOVA followed by Turkey's multiple comparison. Note that the drug concentrations are reported in μM for in vitro experiments and as administered dose (mg/kg) for in vivo experiments, consistent with field standards. [file ACEL-25-e70416-s001.pdf]

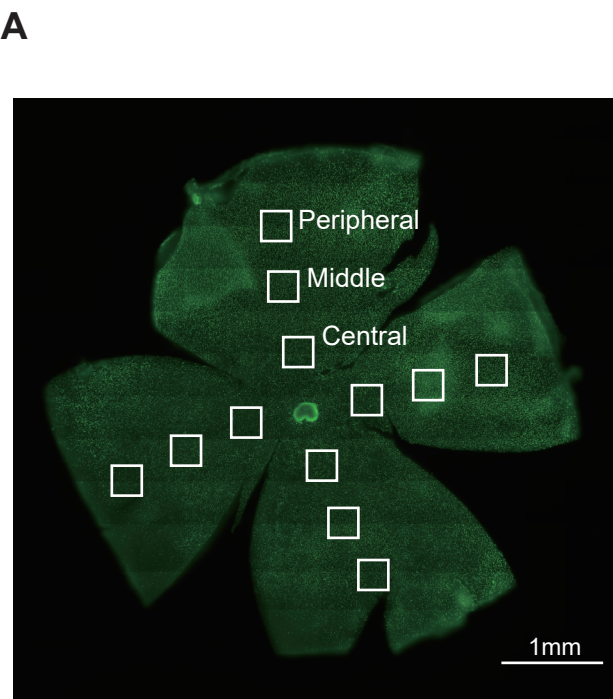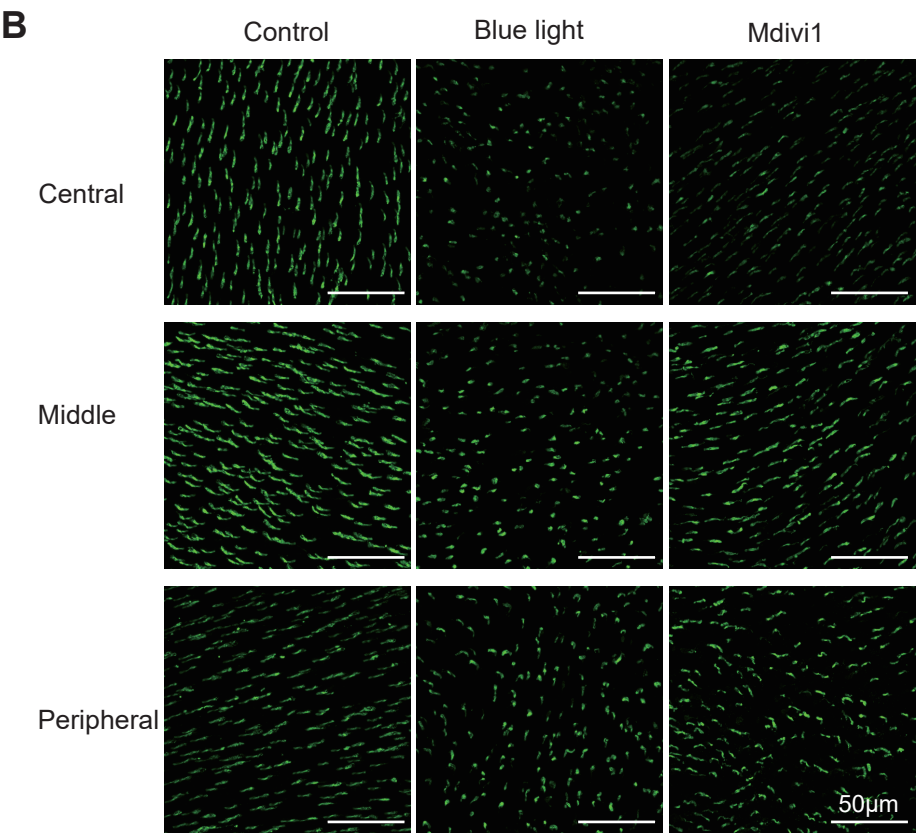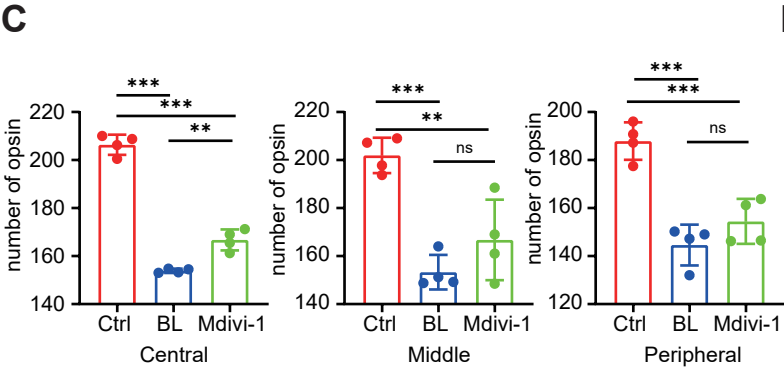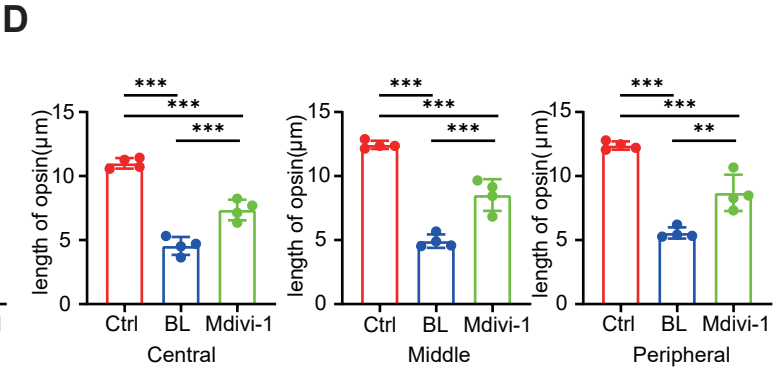

**Supplemental Figure 2**

Supplement: Supplementary file 2 — Figure S2: Mdivi‐1 slows blue light‐induced retinal cone degeneration at various central‐fugal regions. (A) Schematic of the chosen regions across various central‐fugal distances in the experiment. (B) Immunofluorescence images of retinal whole‐mount stained with opsin at various regions. (C, D) The density and length of opsin in the cone outer segment in various regions on whole‐mount retina. **, p < 0.01; ***, p < 0.001; ns, not significantly different; one‐way ANOVA followed by Turkey's multiple comparison. [file ACEL-25-e70416-s003.pdf]
