# Supplementary material for: Dynamin‐Related Protein 1‐Dependent Disruption of Mitochondrial Homeostasis Drives Blue Light‐Induced Epithelial‐Mesenchymal Transition in Retinal Aging
Source: Aging Cell. 2026 Feb 16;25(2):e70416. doi: 10.1111/acel.70416 (PMC12910175; doi:10.1111/acel.70416)

Western blotting supplemental figures

The following are the original membranes for Western blot. The red square indicates the location of the target band in the Western blot.

Fig1F. Drp1 and relative GAPDH.

Used Marker:Thermo Scientific PageRuler 26616

Drp1 82kd

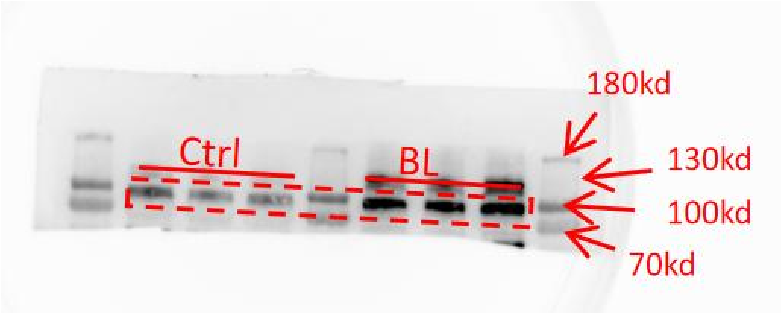

Drp1 82kd （merge with Colorimetric）

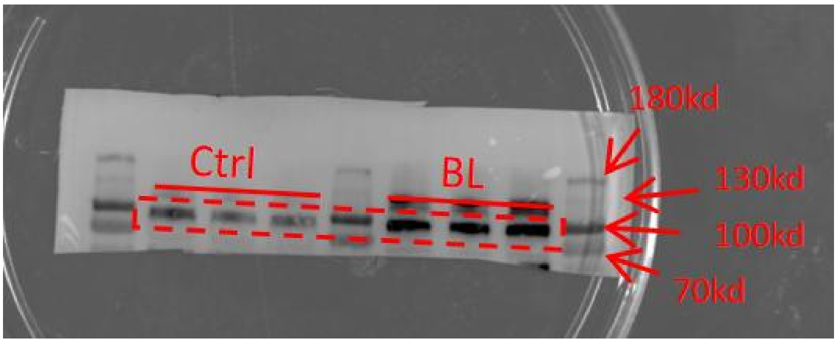

GAPDH 36kd

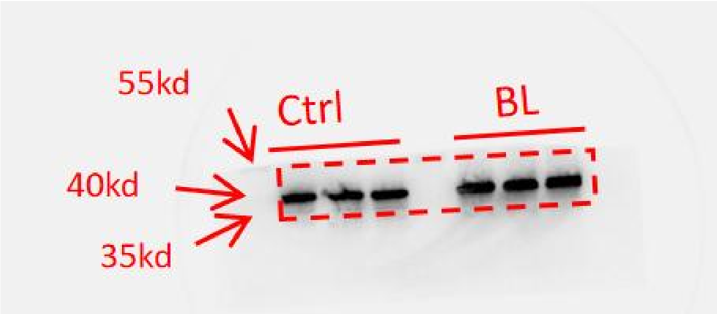

GAPDH 36kd merge with Colorimetric

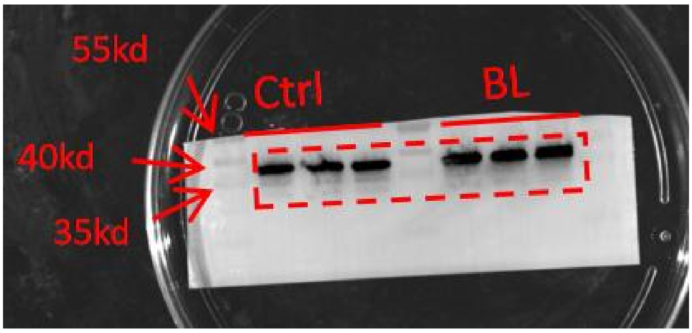

Fig1F. OMA1 and relative β-Actin.

Used Marker:WJ103

OMA1 34kd

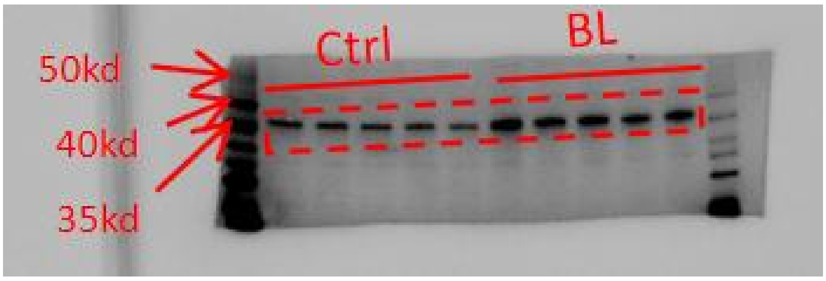

OMA1 34kd merge with Colorimetric

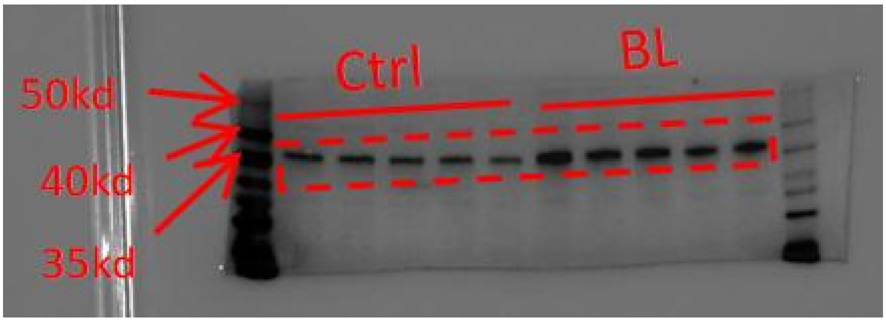

β-Actin 42kd

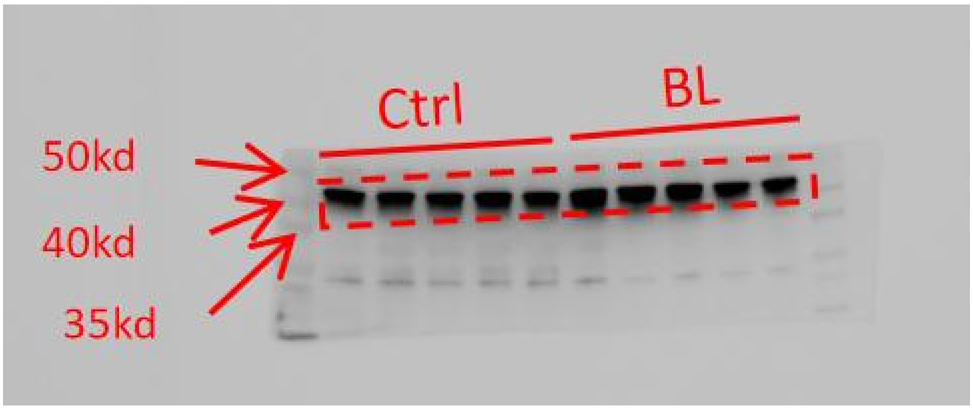

β-Actin 42kd merge with Colorimetric

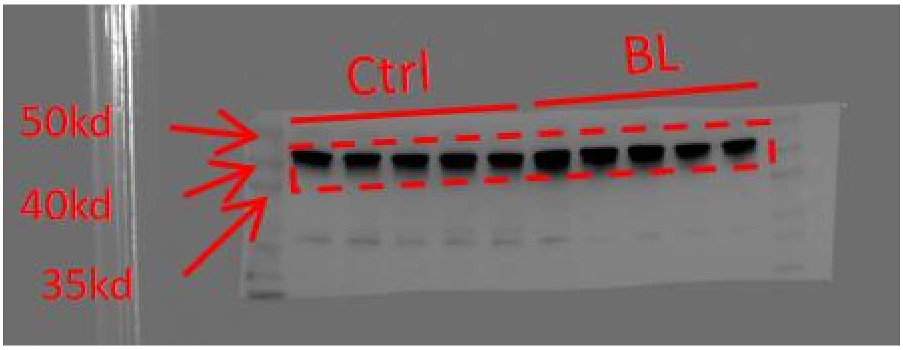

Fig1F. OPA1 and relative β-Actin.
Used Marker:WJ103
OPA1 112kd，Observed :80-100KDa

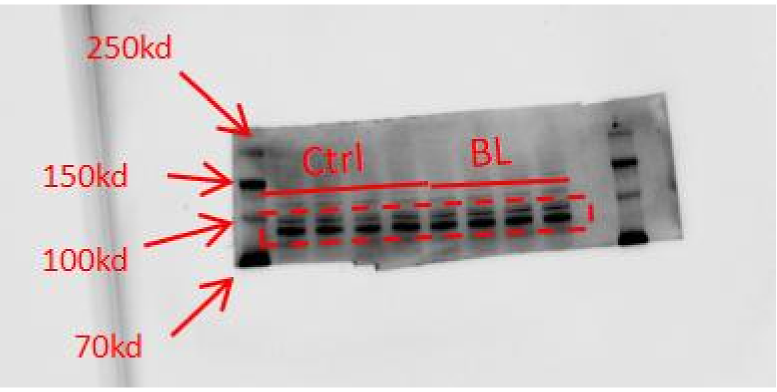

OPA1 merge with Colorimetric

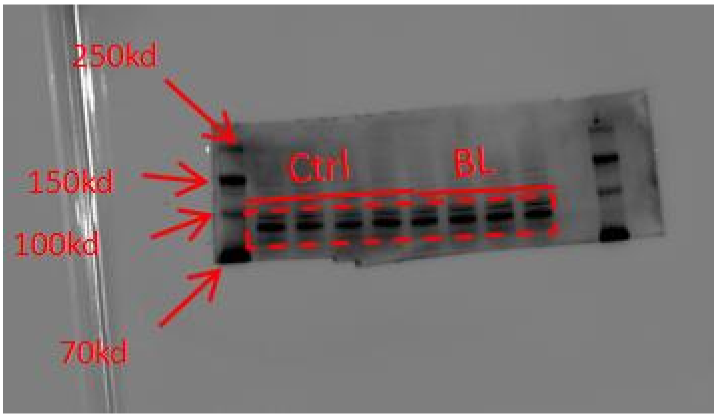

β-Actin 42kd

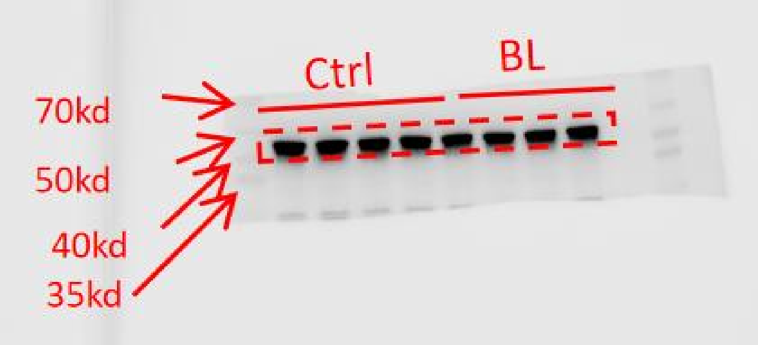

β-Actin 42kd merge with Colorimetric

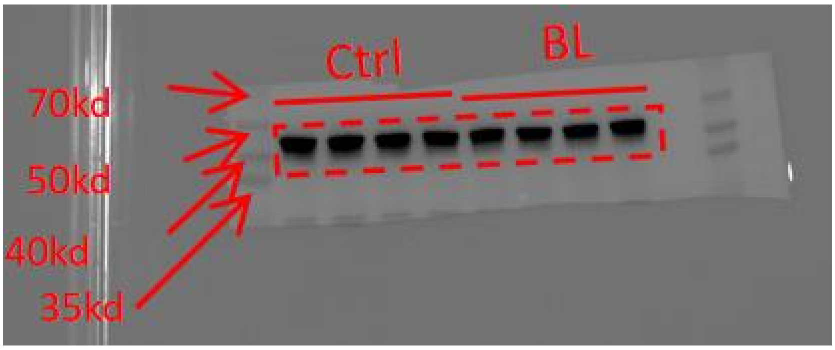

Fig2E. ZO-1 and relative Vinculin.

Used Marker:M5 SuperRange Prestained Protein Ladder (10-310 kDa)

ZO-1 230kd

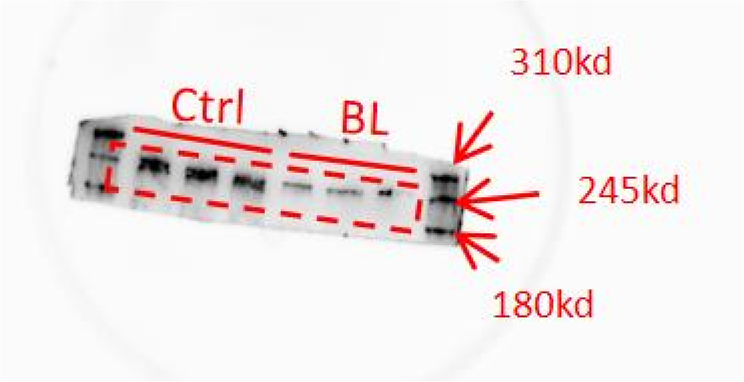

ZO-1 230kd merge with Colorimetric

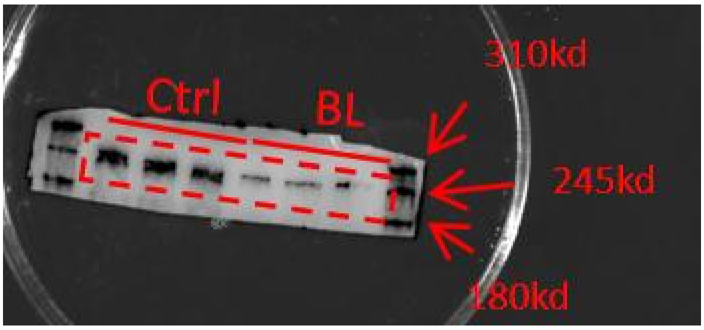

Vinculin 117kd

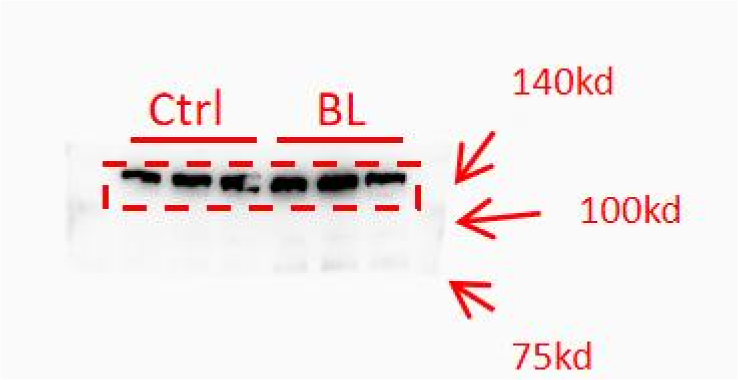

Vinculin 117kd merge with Colorimetric

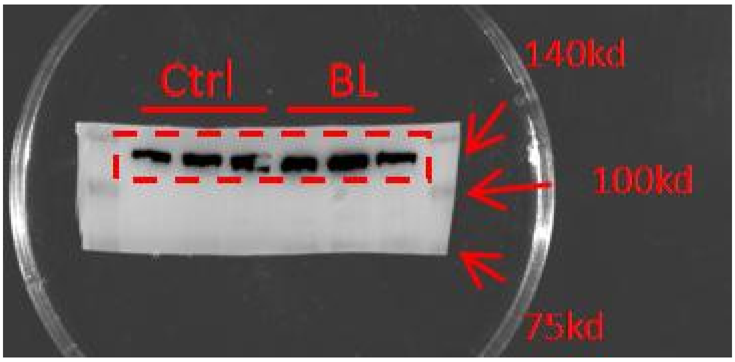

Fig2E. E-Cadherin and relative β-Actin.
Used Marker:WJ103
E-Cadherin 135kd

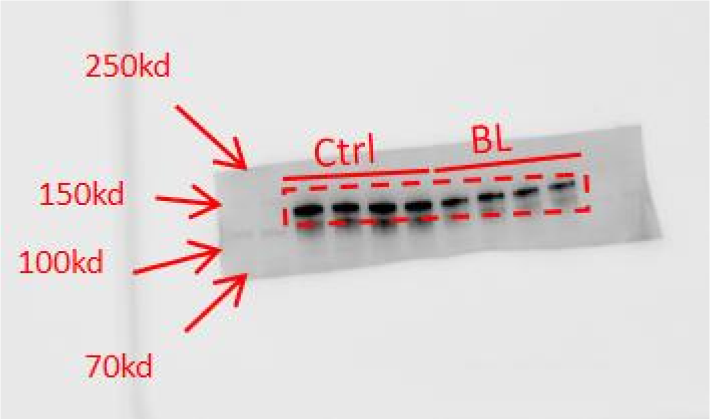

F-Cadherin 135kd merge with Colorimetric

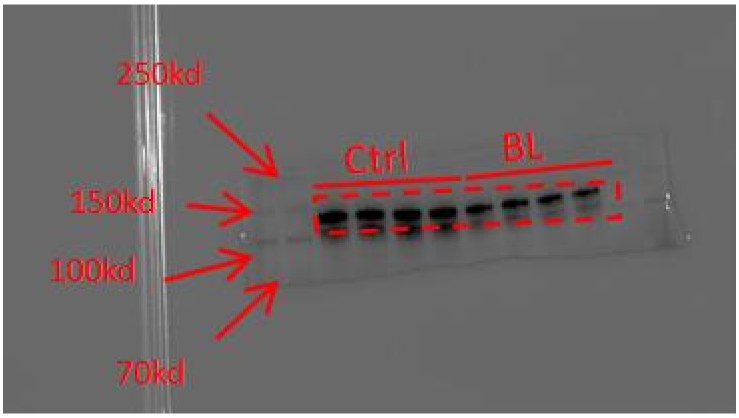

β-Actin 42kd

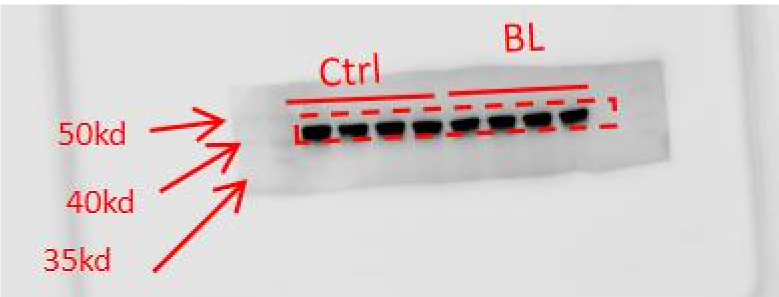

β-Actin 42kd merge with Colorimetric

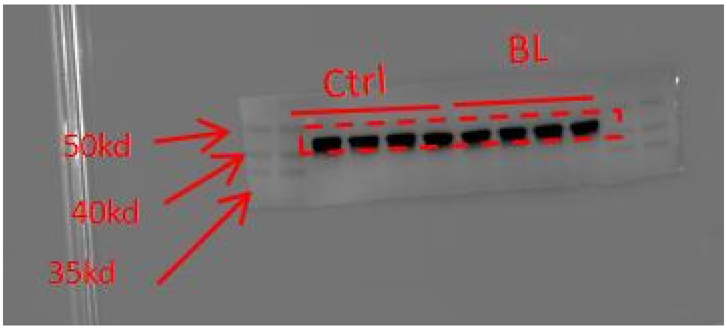

Fig2H. Vimentin and relative GAPDH.

Used Marker:Thermo Scientific PageRuler 26616

Vimentin 54kd

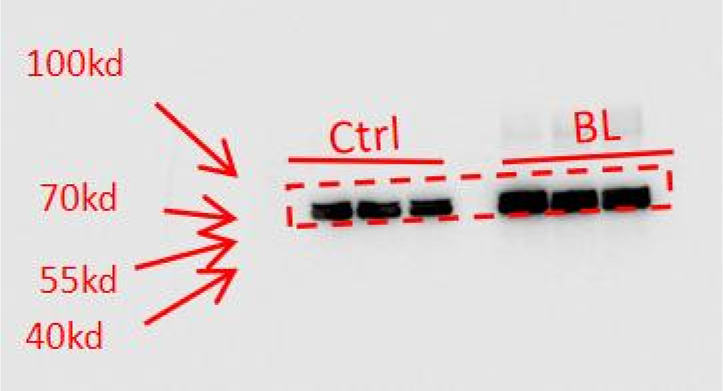

Vimentin 54kd merge with Colorimetric

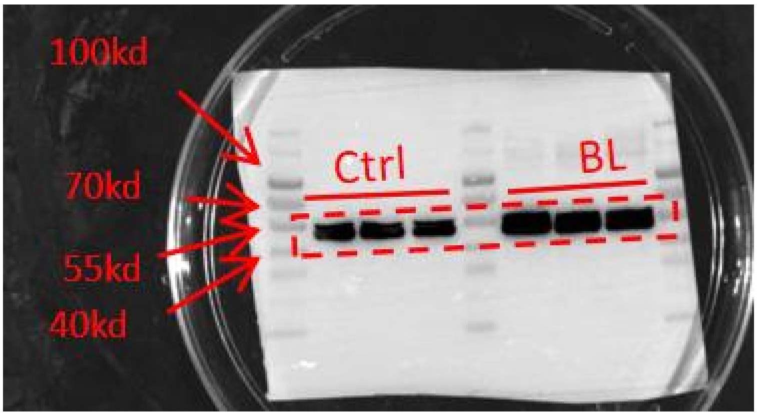

GAPDH 36kd

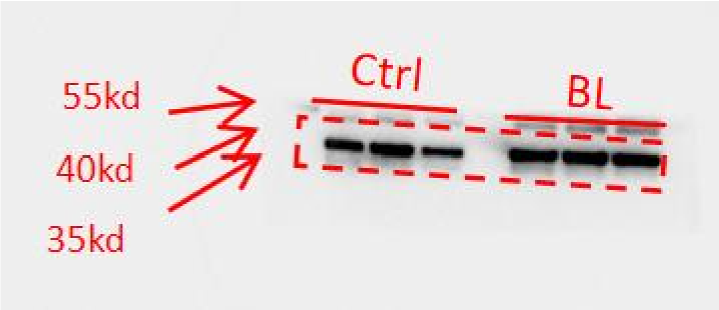

GAPDH 36kd merge with Colorimetric

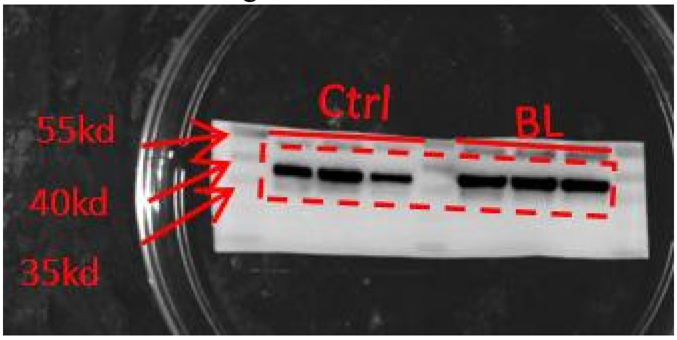

Fig2H. Snail1 and relative β-Tubulin.

Used Marker:Thermo Scientific PageRuler 26616

Snail1 35kd

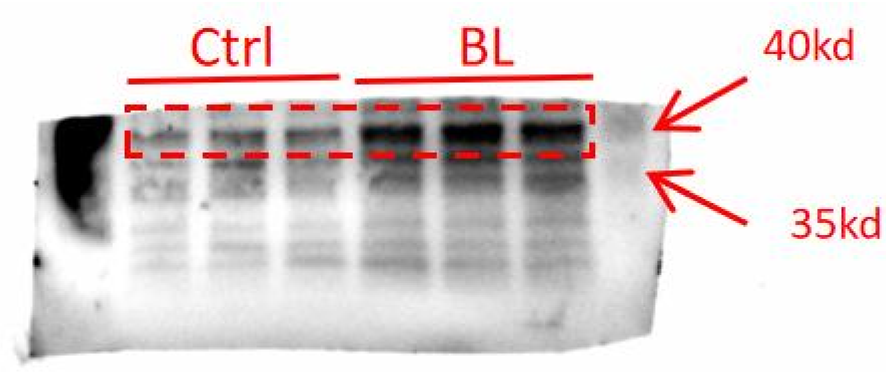

Snail1 35kd merge with Colorimetric

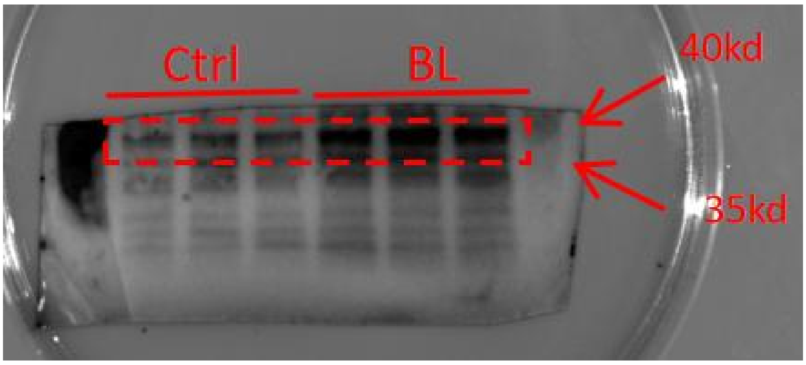

β-Tubulin 55kd

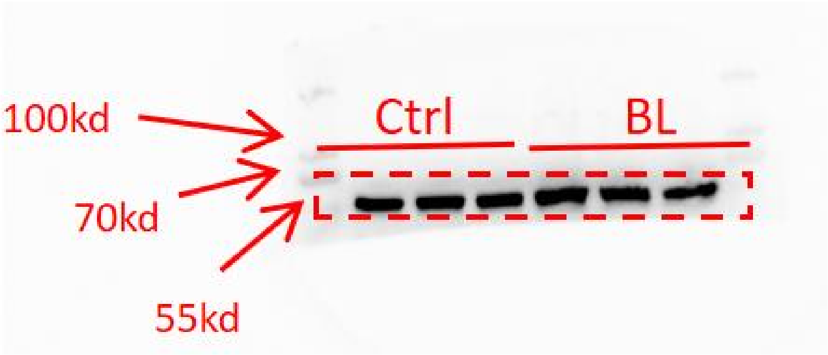

β-Tubulin 55kd merge with Colorimetric

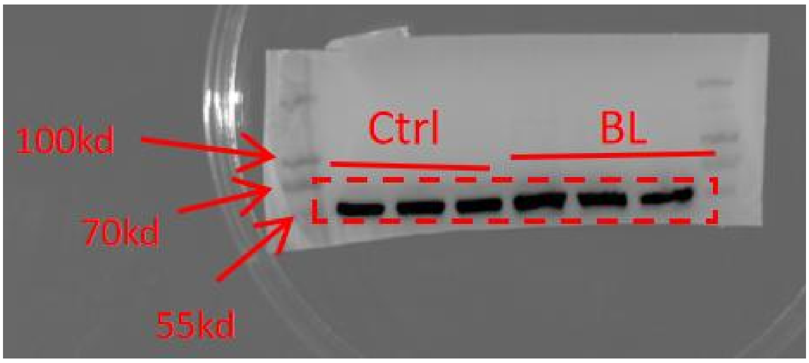

Fig.3D. Drp1 and relative GAPDH.

Used Marker:Thermo Scientific PageRuler 26616

Drp1 82kd

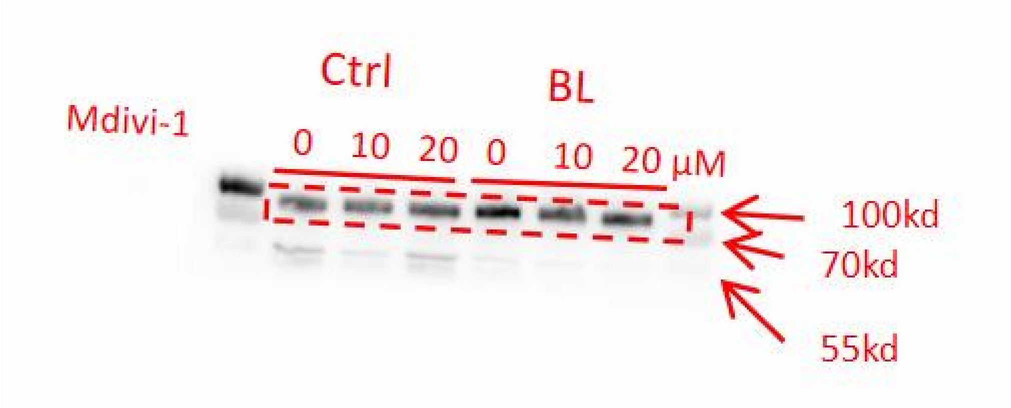

Drp1 82kd merge with Colorimetric

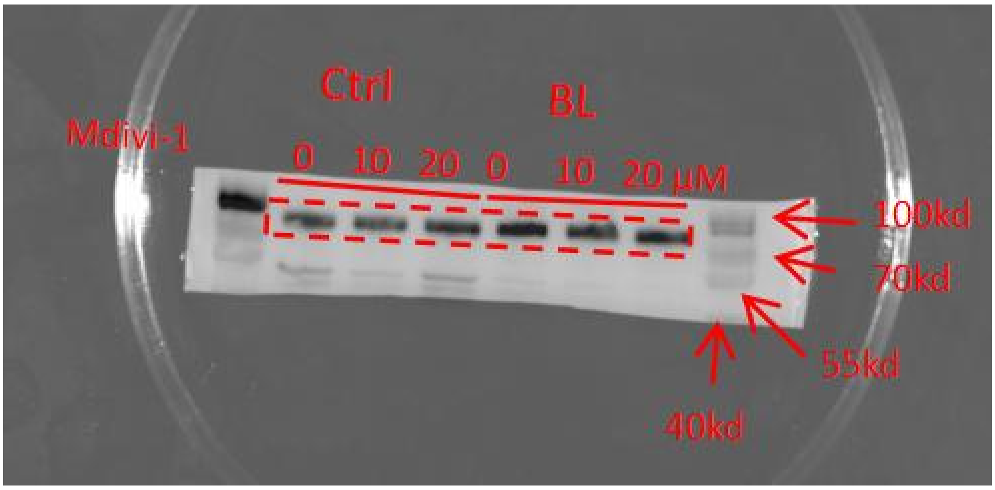

GAPDH 36kd

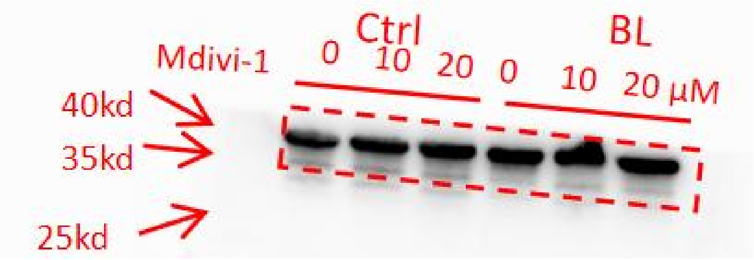

GAPDH 36kd merge with Colorimetric

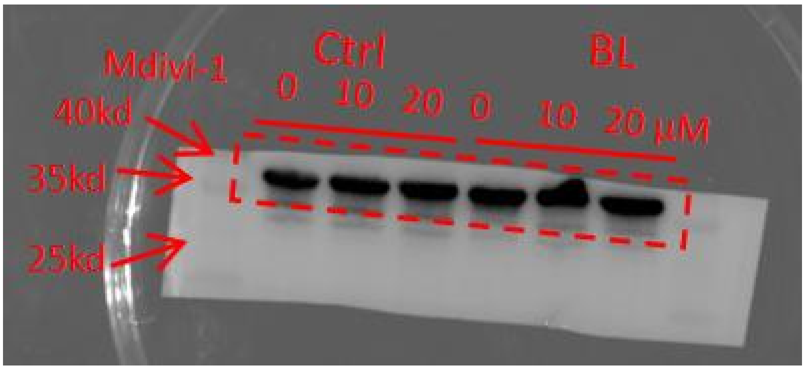

Fig. 3D. MFN2 and relative GAPDH.

Used Marker:Thermo Scientific PageRuler 26616

MFN2 86kd

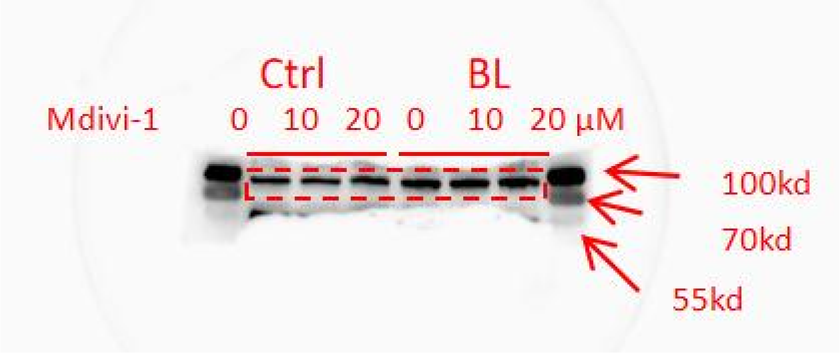

MFN2 86kd merge with Colorimetric

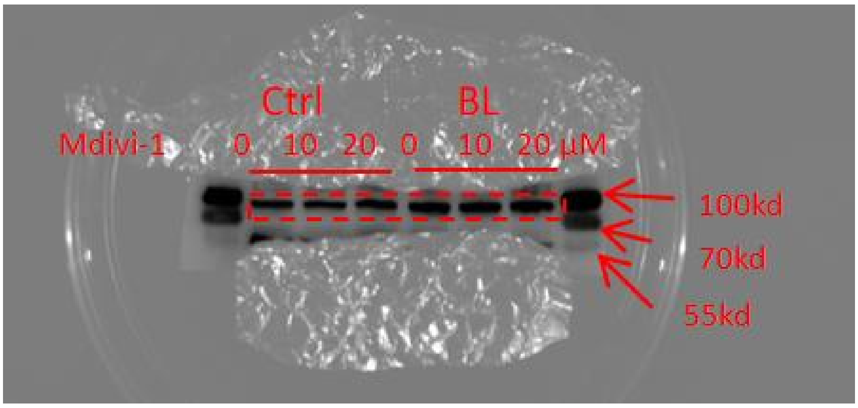

GAPDH 36kd

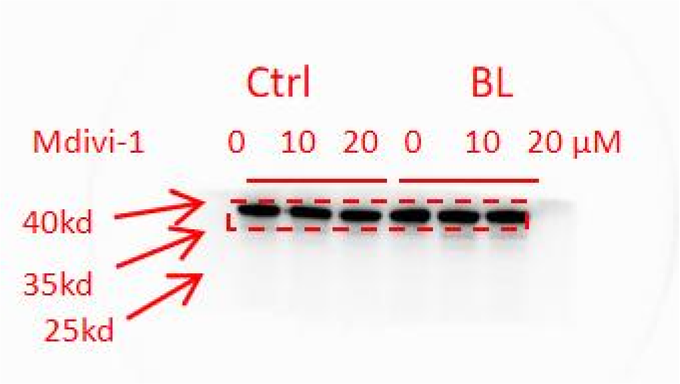

GAPDH 36kd merge with Colorimetric

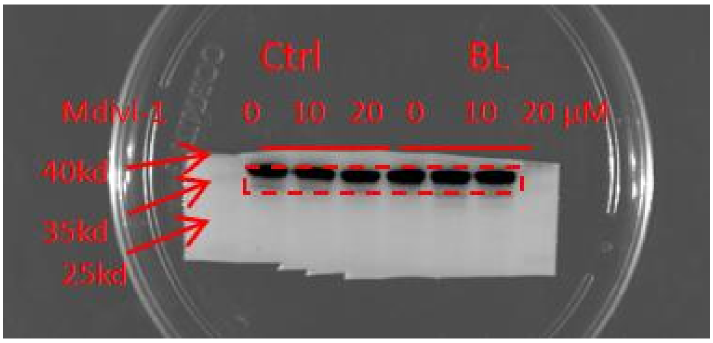

Fig. 3D. OMA1 and relative β-Actin.

Used Marker:WJ103

OMA1 34kd

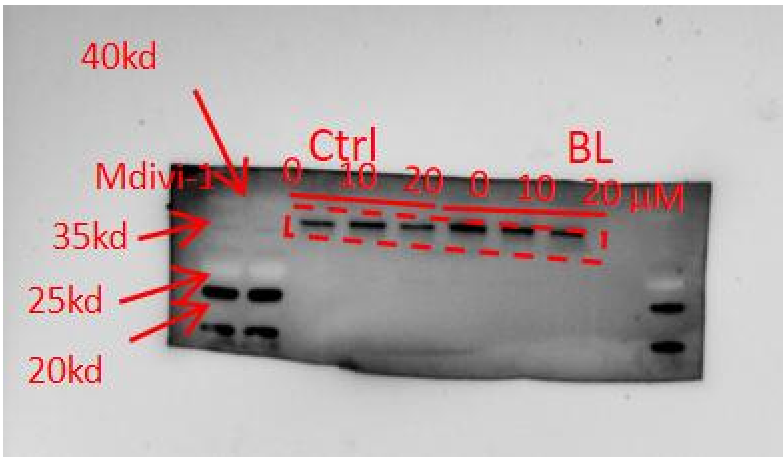

OMA1 34kd merge with Colorimetric

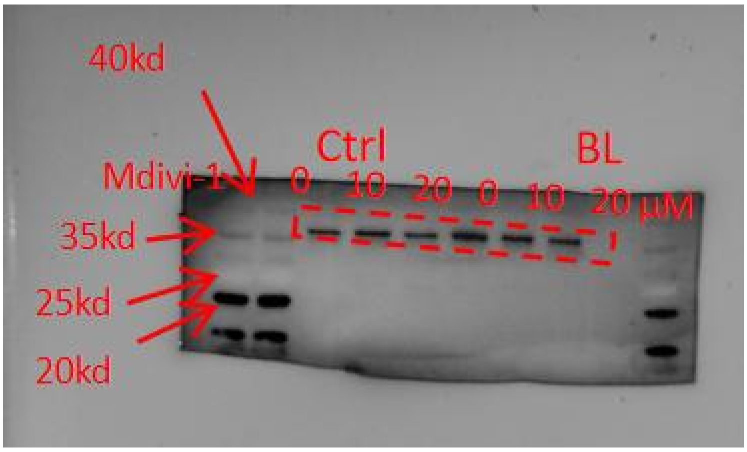

β-Actin 42kd

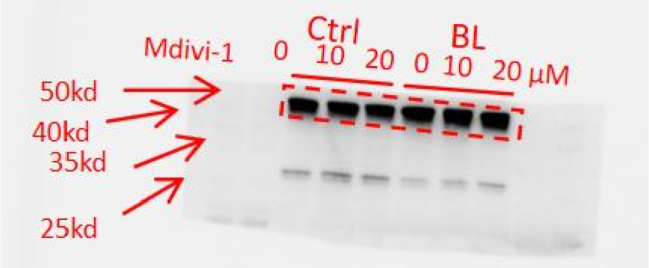

β-Actin 42kd merge with Colorimetric

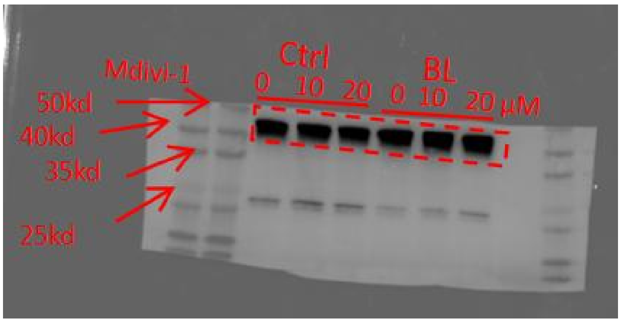

Fig. 3D. OPA1 and relative β-Actin.

Used Marker:WJ103

OPA1 112kd；Observed :80-100KDa

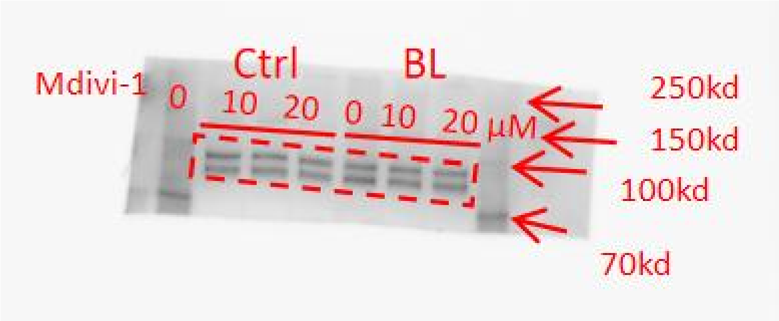

OPA1 112kd；Observed :80-100KDa
merge with Colorimetric

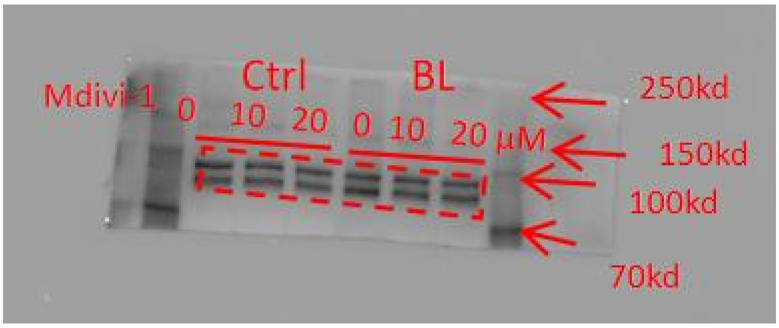

β-Actin 42kd

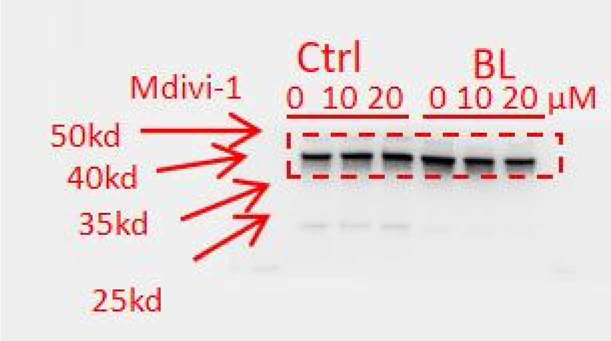

β-Actin 42kd merge with Colorimetric

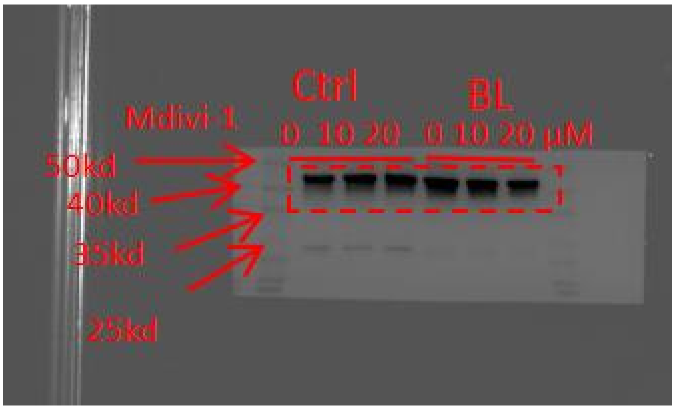

Fig. 4D. E-Cadherin and relative β-Actin.

Used Marker:WJ103

E-Cadherin 135kd

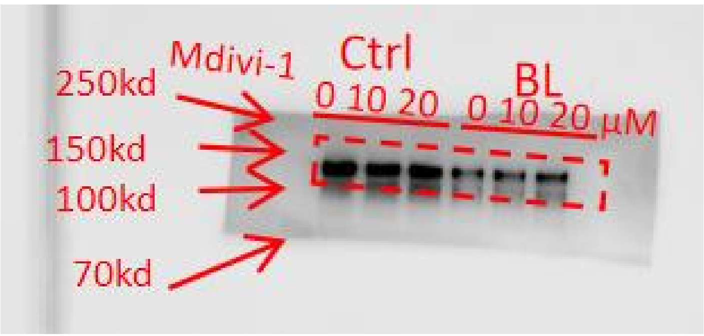

E-Cadherin 135kd merge with Colorimetric

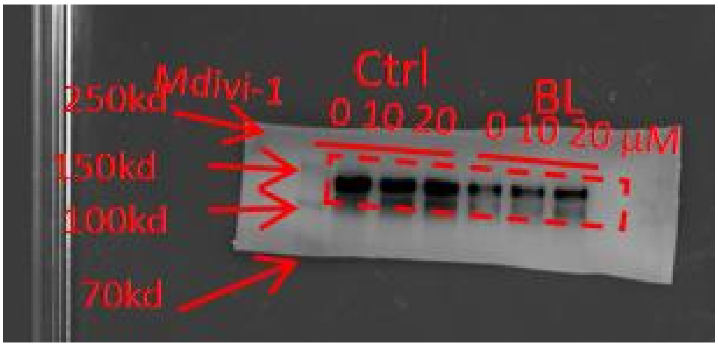

β-Actin 42kd

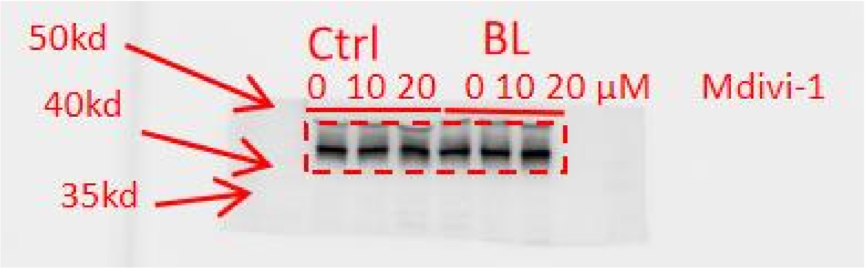

β-Actin 42kd merge with Colorimetric

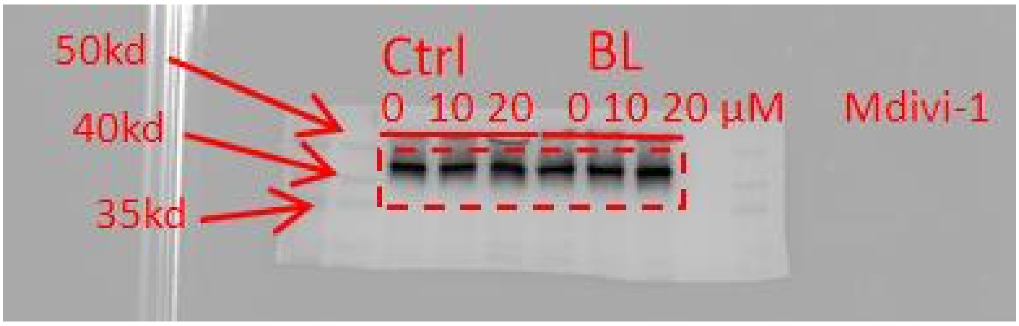

Fig. 4D. ZO-1 and relative Vinculin.

Used Marker:M5 SuperRange Prestained Protein Ladder (10-310 kDa)

ZO-1 230kd

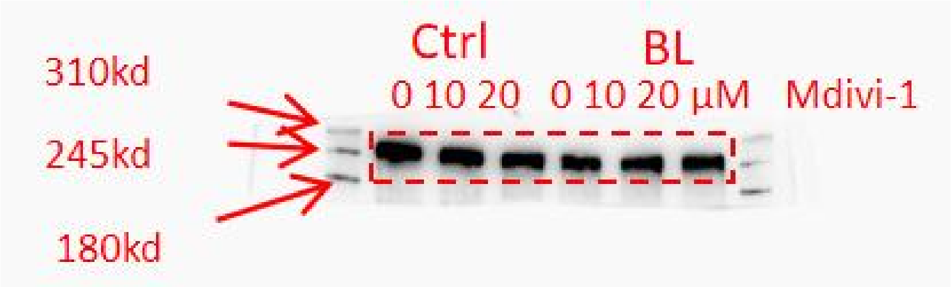

ZO-1 230kd merge with Colorimetric

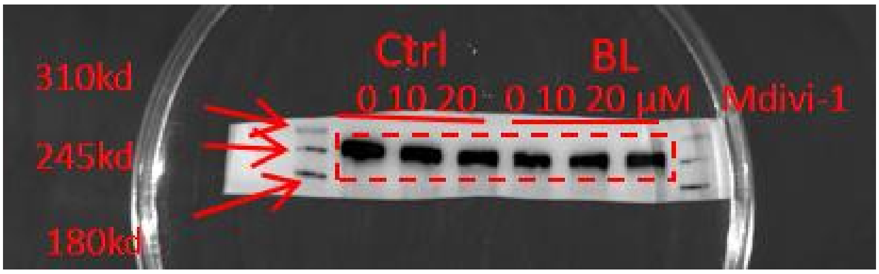

Vinculin 117kd

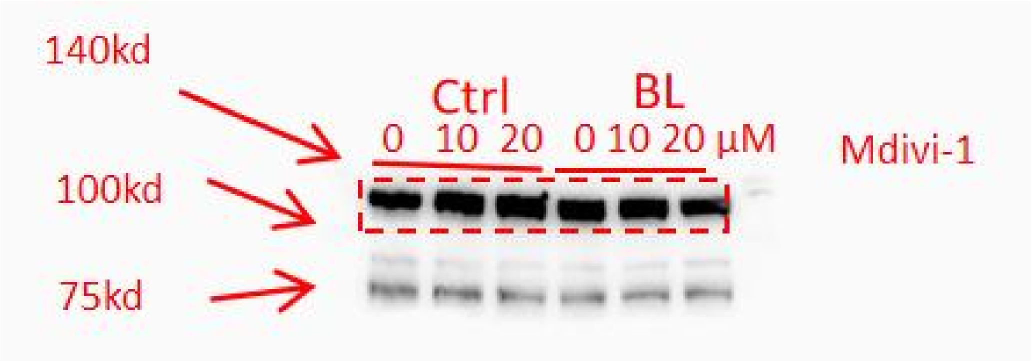

Vinculin 117kd merge with Colorimetric

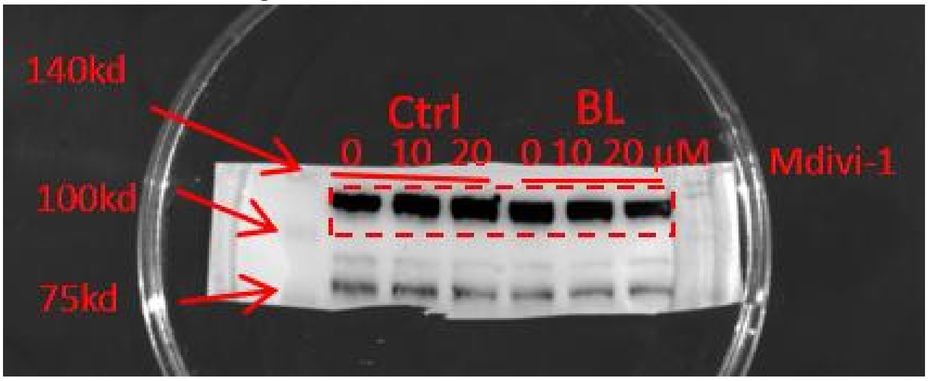

Fig. 4D. Vimentin and relative GAPDH.

Used Marker:WJ103

Vimentin 54kd

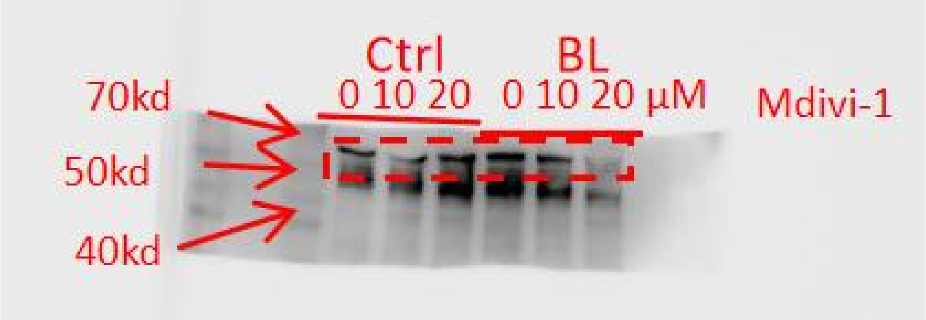

Vimentin 54kd merge with Colorimetric

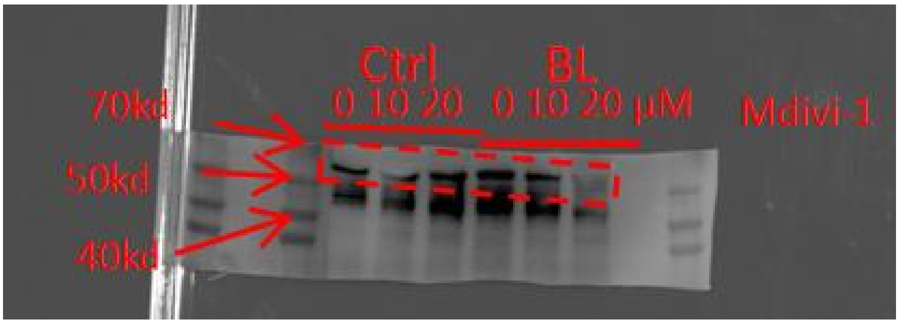

GAPDH 36kd

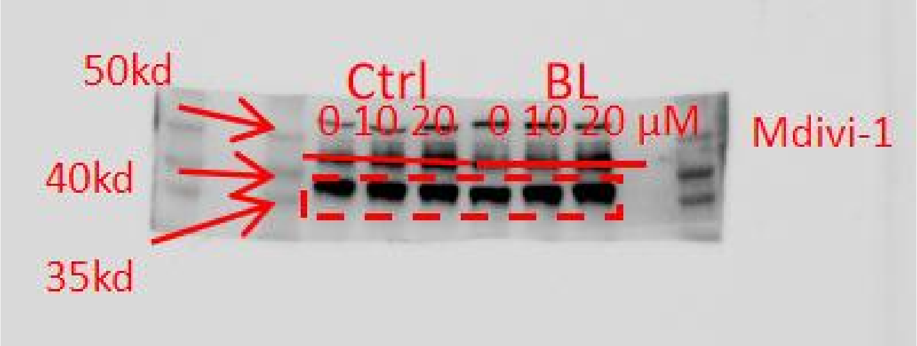

GAPDH 36kd merge with Colorimetric

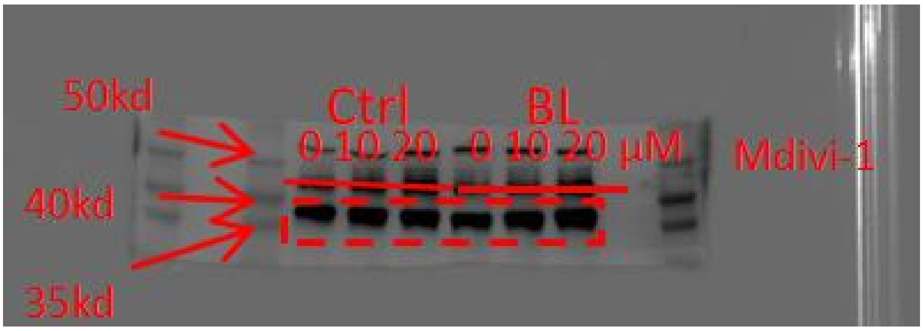

Fig. 4D. Snail1 and relative Vinculin.

Used Marker:Thermo Scientific PageRuler 26616

Snail 1 35kd

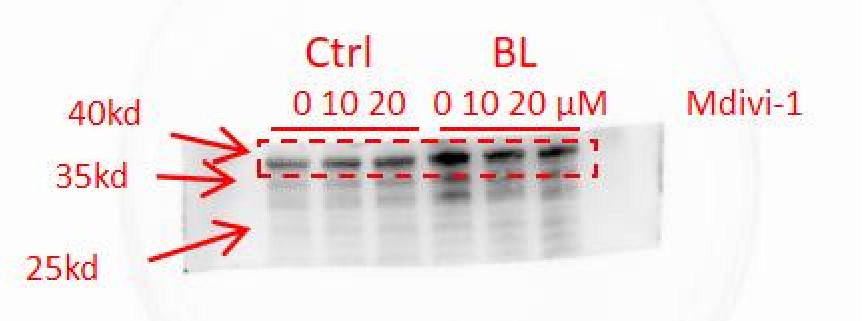

Snail1 35kd merge with Colorimetric

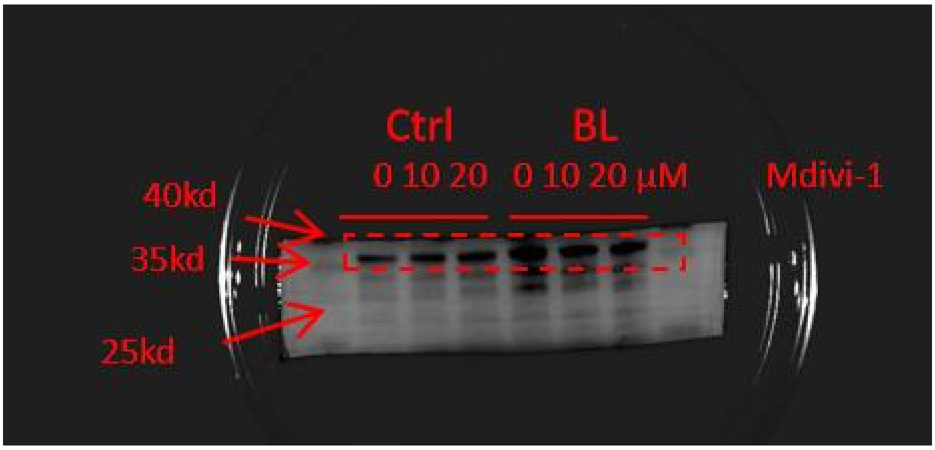

Vinculin 117kd

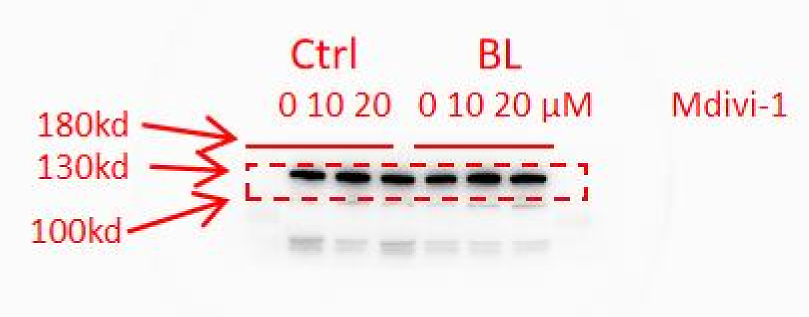

Vinculin 117kd merge with Colorimetric

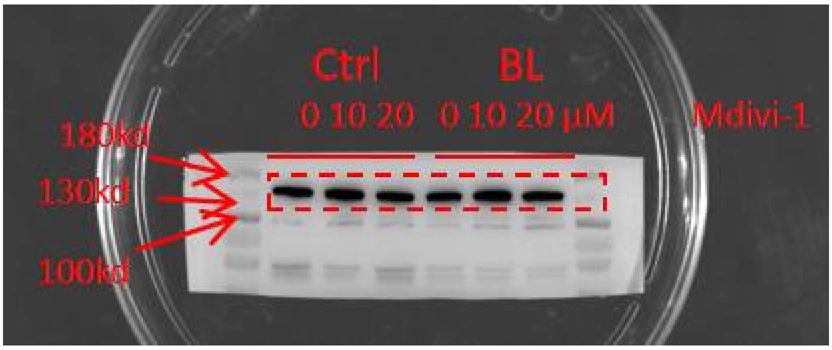

Supplement: Supplementary file 3 — Appendix S1: acel70416‐sup‐0003‐AppendixS1.pdf. [file ACEL-25-e70416-s002.pdf]
